# Tumor Microenvironment Activated Cu Crosslinked Near‐Infrared Sonosensitizers for Visualized Cuproptosis‐Enhanced Sonodynamic Cancer Immunotherapy

**DOI:** 10.1002/advs.202407196

**Published:** 2024-09-27

**Authors:** Jinyan Hu, Lang Yan, Zhi Cao, Bijiang Geng, Xiqian Cao, Bing Liu, Jiaming Guo, Jiangbo Zhu

**Affiliations:** ^1^ Department of Health Toxicology College of Naval Medicine Naval Medical University Shanghai 200433 China; ^2^ Department of Urology Changhai Hospital Naval Medical University Shanghai 200433 China; ^3^ Department of Urology The Third Affiliated Hospital Naval Medical University Shanghai 200433 China; ^4^ Department of Radiation Medicine College of Naval Medicine Naval Medical University Shanghai 200433 China

**Keywords:** cuproptosis, immunotherapy, intelligent nanoassemblies, tumor‐specific disassembly, visualized sonodynamic therapy

## Abstract

Reactive oxygen species (ROS)‐mediated sonodynamic therapy (SDT) holds increasing potential in treating deep‐seated tumor owing to the high tissue‐penetration depth. However, the inevitable accumulation of sonosensitizers in normal tissues not only make it difficult to realize the in situ SDT, but also induces sonodynamic effects in normal tissues. Herein, this work reports the passivation and selective activation strategies for the sonodynamic and near‐infrared (NIR) imaging performances of an intelligent antitumor theranostic platform termed Cu‐IR783 nanoparticles (NPs). Owing to the ruptured coordination bond between IR783 with Cu ions by responding to tumor microenvironment (TME), the selective activation of IR783 only occurred in tumor tissues to achieve the visualized in‐situ SDT. The tumor‐specific released Cu ions not only realized the cascade amplification of ROS generation through Cu^+^‐mediated Fenton‐like reaction, but also triggered cuproptosis through Cu^+^‐induced DLAT oligomerization and mitochondrial dysfunction. More importantly, the immunosuppressive TME can be reversed by the greatly enhanced ROS levels and high‐efficiency cuproptosis, ultimately inducing immunogenic cell death that promotes robust systemic immune responses for the eradication of primary tumors and suppression of distant tumors. This work provides a distinct paradigm of the integration of SDT, CDT, and cuproptosis in a controlled manner to achieve visualized in‐situ antitumor therapy.

## Graphical abstract

The passivation and selective activation strategies for the sonodynamic and NIR imaging performances of NIR sonosensitizers are reported. An intelligent antitumor theranostic platform termed Cu‐IR783 is facilely constructed. The tumor‐specific released Cu ions realize the cascade amplification of ROS generation and triggeres cuproptosis. ICD effects are induced by enhanced ROS levels and cuproptosis, which promote robust systemic immune responses.

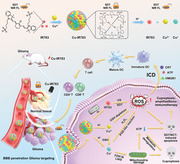

## 1 Introduction

Although traditional therapeutic methods such as surgery, chemotherapy, and radiotherapy show a certain positive effect on glioblastoma, problems such as tumor recurrence and metastasis are prominent.^[^ ^1^, ^2^, ^3^ ^]^ Recently, the development of nanomedicine has provided possibilities for overcoming drug resistance, improving tumor targeting, and reducing toxic side effects.^[^ ^4^, ^5^ ^]^ For example, nanotechnology utilizing reactive oxygen species (ROS) for combating cancer has received widespread attention.^[^ ^6^ ^]^ ROS is a double‐edged sword in the process of tumor progression, in which low levels of ROS are involved in the occurrence and development of tumors, while high levels of ROS initiate oxidative stress responses in cancer cells and lead to cell apoptosis.^[^ ^7^ ^]^ Some novel tumor treatment modalities that utilize exogenous stimuli or endogenous chemical reactions to generate ROS have been widely studied, including photodynamic therapy (PDT),^[^ ^8^, ^9^, ^10^ ^]^ sonodynamic therapy (SDT),^[^ ^11^, ^12^, ^13^, ^14^ ^]^ and chemodynamic therapy (CDT).^[^ ^15^, ^16^, ^17^ ^]^ Among them, SDT can overcome the inherent defects of PDT owing to the higher tissue penetration depth (>10 cm).^[^ ^18^, ^19^, ^20^ ^]^ Moreover, the cavitation effect can promote the vascular penetration of nanodrugs into the interior of tumors, especially raising the permeability of the blood‐brain barrier (BBB) to enhance the therapeutic effects of GBM.^[^ ^21^, ^22^ ^]^

By analyzing the mechanism of SDT, the efficacy of SDT largely depends on the performance of sonosensitizers and its regulation of the tumor microenvironment (TME).^[^ ^23^ ^]^ To overcome the limitations of inorganic sonosensitizers represented by TiO_2_, many strategies have been explored to reduce the bandgap of TiO_2_ by introducing oxygen vacancies or doping metal ions and suppressing the recombination of electron‐hole pairs through the construction of heterojunctions with matched bandgap structures.^[^ ^24^, ^25^, ^26^, ^27^ ^]^ In response to the constraints of TME, nanozymes with multiple enzyme‐mimic catalytic activities were introduced into the sonosensitizer system, which not only consumes overexpressed glutathione (GSH) but also alleviates severe hypoxia, thereby achieving cascade amplification of ROS production.^[^ ^28^, ^29^, ^30^ ^]^ On this basis, further introduction of nanozymes with peroxidase (POD)‐mimic catalytic activity could produce hydroxyl radical (•OH) through endogenous Fenton/Fenton‐like reactions, realizing the combination of exogenous SDT and endogenous CDT.^[^ ^31^, ^32^, ^33^ ^]^ Although multiple combination therapy strategies can enhance the efficacy of SDT and achieve a “1+1>2” therapeutic effect, SDT still faces the following challenges that need to be overcome.

First, the development of sonosensitizers commonly only focuses on improving the ROS generation efficiency, while neglecting the focus on biocompatibility of sonosensitizers, which could lead to potential safety issues.^[^ ^34^ ^]^ Second, the systemically distributed sonosensitizers without active targeting capabilities after intravenous administration would also induce sonodynamic effects in normal tissues, causing irreversible damage to normal tissues or organs.^[^ ^35^ ^]^ Thirdly, it is difficult to visually monitor whether the sonosensitizers can accumulate in tumors during the SDT process. To solve these above problems, it is a significant challenge to design a smart sonosensitizer that the sonodynamic activity and imaging ability of passivated sonosensitizers and fluorescent probes can only be activated in TME to achieve the visualized in‐situ SDT.

In addition to SDT, metal‐ions‐mediated tumor therapy has also received widespread attention such as cuproptosis, ferroptosis, pyroptosis, and metalloimmunotherapy.^[^ ^36^, ^37^, ^38^, ^39^, ^40^, ^41^, ^42^ ^]^ Biological systems typically regulate Cu ions levels through evolutionarily conserved homeostatic mechanisms, but an excessive amount of Cu ions can be toxic and lead to cell death.^[^ ^43^, ^44^, ^45^, ^46^, ^47^ ^]^ The proposal mechanism of cuproptosis is mainly ascribed to that the excessive Cu ions could induce the aggregation of lipoylated proteins and inhibit mitochondrial metabolic function by binding with lipoprotein, eventually triggering cell death.^[^ ^37^, ^48^, ^49^, ^50^, ^51^, ^52^ ^]^ By analyzing the mechanism of cuproptosis, it is not difficult to find that how to improve the accumulation level of Cu ions in tumor cells is the key factor affecting the therapeutic effects. Owing to the limited intracellular concentration of Cu ions, the frequently drug administration need to be performed to improve the accumulation level of Cu ions in tumor tissues for the enhancement of therapeutic effects.^[^ ^53^, ^54^ ^]^ However, similar to SDT, cuproptosis‐induced cell death did not possess high‐selectivity, resulting that the systemically distributed Cu ions would also induce cuproptosis in normal tissues.^[^ ^55^, ^56^ ^]^ Therefore, it is urgent to develop multifunctional intelligent nanosystems involved sonosensitizers and Cu ions, which can not only realize the passivation of sonodynamic activity, but also achieve the TME‐responsive release of sonosensitizers and Cu ions.

In this work, we reported for the first time the construction of intelligent nanoassemblies termed Cu‐IR783 nanoparticles (NPs) with passivated sonodynamic activity and NIR imaging ability based on the coordination ability of Cu^2+^ and IR783 sonosensitizers. As a commercial NIR imaging agent, IR783 could serve as a theranostic NIR sonosensitizer,^[^ ^57^, ^58^ ^]^ however, the free IR783 with the systemically distributed features cannot realize the in‐situ and visualized SDT owing to the short blood circulation time and limited tumor accumulation.^[^ ^59^ ^]^ After the simple assembly process, the obtained Cu‐IR783 NPs exhibited higher tumor accumulation compared to the free IR783 owing to the EPR effect induced by the increased particle size. While the NIR imaging and sonodynamic capabilities of Cu‐IR783 NPs were passivated under the normal physiological conditions, the selective activation for NIR imaging, SDT, and CDT only occurred in tumor tissues owing to the ruptured coordination bond between IR783 with Cu ions in an acidic TME, consequently avoiding the side effects on normal tissues. Moreover, the deep tumor penetration was achieved by the TME‐responsive disassembly of Cu‐IR783 owing to the smaller size of the released IR783 and Cu ions, manifesting the intelligent of nanoassemblies with size‐morphing capability. The TME‐responsive released IR783 achieved the visualized in‐situ SDT owing to the activated NIR imaging ability and sonodynamic activity. In addition, the released Cu ions not only realized the cascade amplification of ROS generation through Cu^+^‐mediated Fenton‐like reaction, but also triggered tumor‐specific cuproptosis through Cu^+^‐induced DLAT oligomerization and mitochondrial dysfunction. More importantly, the greatly improved ROS level and efficient cuproptosis caused by TME‐activated Cu‐IR783 NPs reversed the immunosuppressive TME and triggered ICD to stimulate systemic immune responses. Owing to these favorable features, the integration of SDT, CDT, and cuproptosis in a controlled manner could achieve an intelligent, visualized, and in‐situ anticancer strategy with minimized sides effects.

## 2 Results and Discussion

### 2.1 Preparation and Characterization of Cu‐IR783 NPs

**Scheme 1:**
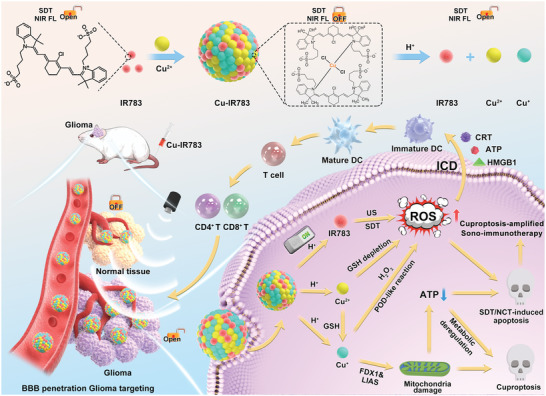
Schematic illustration of the preparation of intelligent nanoassemblies termed Cu‐IR783 NPs with “off/on” function of sonodynamic activity and NIR imaging ability, and mechanism of the visualized in‐situ SDT/CDT combined with cuproptosis for cancer theranostic.

**Figure 1:**
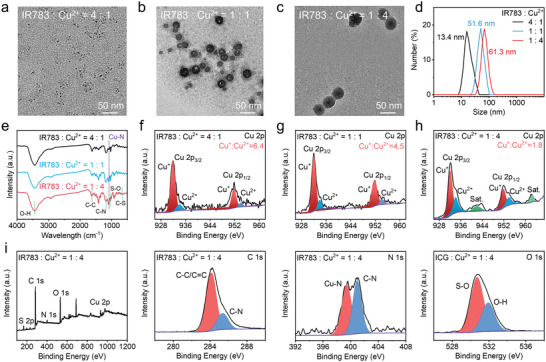
Characterization of Cu‐IR783. a–e) TEM images (a–c), hydrodynamic diameter (d), and FTIR spectra (e) of nanoassemblies at different feeding ratio of IR783 to Cu^2+^. f–h) High‐resolution Cu 2p spectra of nanoassemblies at different feeding ratio of IR783 to Cu^2+^. i) Survey XPS, high‐resolution C 1s, N 1s, O 1s, and S 2p spectra of nanoassemblies at IR783 to Cu^2+^ ratio of 1:4.

The construction of Cu^2+^‐induced IR783 assembly (Cu‐IR783 NPs) is depicted in **Scheme** **1**. By changing the feeding concentrations of IR783 and Cu^2+^ (4:1, 1:1, and 1:4), three kinds of Cu‐IR783 NPs were prepared for the subsequent study of structure and properties. In this design, the NIR sonosensitizer IR783 with efficient NIR fluorescence imaging ability is used for imaging‐guided SDT. Cu^2+^ is introduced to assemble IR783 for the passivation of sonodynamic activity and imaging ability of IR783. We initially utilized TEM to demonstrate the assembly of Cu^2+^ and IR783 into nanoassemblies. **Figure** **1**a–c exhibited that these Cu‐IR783 NPs were found to be well‐dispersed sphere NPs. The size distribution of the three kinds of Cu‐IR783 was then measured by dynamic light scattering (DLS), which indicated that the particle size of Cu‐IR783‐1, Cu‐IR783‐2, and Cu‐IR783‐3 was determined to be ≈ 13.4, 51.6, and 61.3 nm, respectively (Figure 1d). The size distribution of Cu‐IR783, when compared to free IR783, facilitated enhanced accumulation in tumor tissue.

Subsequently, we performed FTIR and XPS to investigate the chemical components and surface structure of these Cu‐IR783 NPs. The obvious characteristic peaks at ≈650, 1020, 1120, 1650, and 3450 cm^−1^ can be detected in the FTIR spectra of the three kinds of Cu‐IR783 (Figure 1e), which corresponded to C─S, S─O, C─N, C─C, and O─H stretching vibrations, respectively. The presence of C─S, S─O, C─N ascribed to IR783 in these Cu‐IR783 NPs demonstrated the successful fabrication of nanoassemblies. The existence of Cu─N at ≈ 1045 cm^−1^ was observed in the FTIR spectra of the three kinds of Cu‐IR783, suggesting the coordination between Cu^2+^ and IR783. Moreover, the strength of Cu─N bond increased as the feeding concentrations of Cu^2+^, demonstrating that the increase of Cu^2+^ could induce more IR783 assembly. The survey XPS spectra of the three kinds of nanoassemblies exhibited the presence of Cu 2p, C 1s, N 1s, O 1s, and S 2p peaks (Figure 1i; Figures S2, and S3, Supporting Information), confirming the presence of Cu ions and IR783. For the high‐resolution Cu 2p spectrum, the satellite peaks of Cu^+^ and Cu^2+^ were detected in the three kinds of Cu‐IR783 (Figure 1f–h), elucidating that partial Cu^2+^ have been reduced to Cu^+^ by IR783. In addition, the ratio of Cu^+^ to Cu^2+^ was decreased from 6.4 to 1.8 as the feeding concentration of Cu^2+^ increasing, which could be ascribed to the saturation of the coordination between Cu^2+^ and IR783. The appearance of Cu^+^ in nanoassemblies after assembly could be attributed to the reduction of Cu^2+^ by IR783, which was similar to the previous reports.^[^ ^60^, ^61^ ^]^ Furthermore, the presence of C─C/C═C, C─N, Cu─N, C─N, S─O, and O─H can be detected in the high‐resolution C 1s, N 1s, O 1s spectra of these Cu‐IR783 NPs (Figures S1 and S2, Supporting Information), clearly verifying the successful preparation of self‐assembly Cu‐IR783. We also measured the content of Cu ions in Cu‐IR783 prepared by different feeding ratios (IR783: Cu = 4:1, 1:1, or 1:4) using ICP‐MS analysis. The loading efficiency of Cu ions in Cu‐IR783 prepared by different feeding ratios (IR783: Cu = 4:1, 1:1, or 1:4) was determined to be ≈4.55%, 7.13%, or 9.05%, respectively.

### 2.2 Cu^2+^‐Induced Passivation Effects of IR783

**Figure 2:**
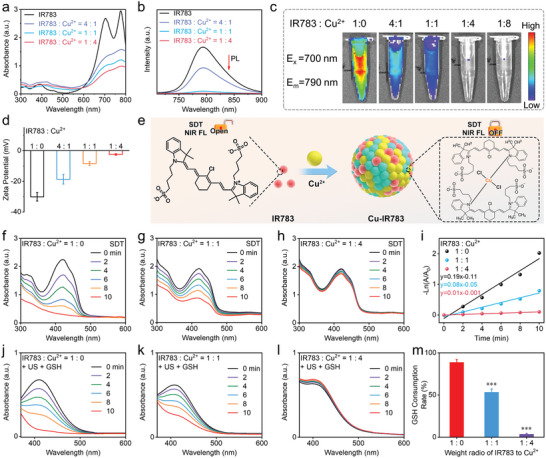
Cu^2+^‐Induced Passivation Effects of IR783. a–d) Absorption spectra (a), NIR fluorescence spectra (b), NIR images (c), and Zeta potential (d) of nanoassemblies at the varied feeding concentrations of Cu^2+^ and IR783. e) Schematic illustration of the synthesis of nanoassemblies for the passivation of sonodynamic activity and NIR imaging ability of IR783. f–h) Absorption spectra of the free IR783 and nanoassemblies under US irradiation. i) Comparison of the sonodynamic activity of the free IR783 and nanoassemblies. j–l) Absorption spectra of the free IR783 and nanoassemblies in the presence of GSH and US irradiation. m) Comparison of the GSH depletion ability of the free IR783 and nanoassemblies. Data are presented as the mean ± SD. (n = 3).

Having confirmed the successful assembly of Cu^2+^ and IR783, we then investigated the influence of assembly on the sonodynamic activity and NIR imaging capability of the free IR783. The characteristic absorption peaks of IR783 located at ≈700 and 780 nm (**Figure** **2**a). The decreased intensity of the two peaks in IR783 solution was detected after the addition of Cu^2+^. In addition, the continuous decrease of peak intensity was observed after the concentration of Cu^2+^ increasing (Figure 2e). More interestingly, the fluorescence intensity of IR783 at the optimum emission peak (≈800 nm) exhibited a significant decrease after being assembled to Cu‐IR783 NPs (Figure 2b), which could be attributed to the aggregation of IR783 induced by Cu^2+^ and the subsequent electron/energy transfer mechanisms. It should be noted that the completely quenched fluorescence intensity of IR783 was detected at the IR783/Cu^2+^ ratio of 1: 4. We also monitored the NIR fluorescence change of IR783 after adding Cu^2+^. As depicted in Figure 2c, the gradually quenched NIR fluorescence of IR783 was observed after the assembled to Cu‐IR783 NPs. The fluorescence imaging ability of IR783 completely disappeared at the IR783/Cu^2+^ ratio of 1: 4, which was consistent with the fluorescence spectrum results. Apart from the decrease of fluorescence intensity, the fluorescence lifetime of IR783 was also decreased after being assembling to Cu‐IR783 NPs, which was decreased from 960.56 to 101.96 ns at the IR783/Cu^2+^ ratio of 1: 4 (Figure S4, Supporting Information). The gradually increased Zeta potential of nanoassemblies was observed as the feeding concentrations of positively charged Cu^2+^ increasing (Figure 2d), demonstrating the successful assembly of Cu‐IR783 NPs.

The passivation effects of Cu^2+^ on the sonodynamic activity of IR783 were then investigated. The efficient sonodynamic activity of IR783 was first demonstrated (Figure 2f), which revealed that the characteristic absorption peak of DPBF was significantly decreased after the treatment of US irradiation for 10 min. The rate constant of ROS (^1^O_2_ or O_2_^−•^) generation in the presence of the free IR783 was determined to be 0.19 min^−1^ (Figure 2i). We also investigated that whether the IR783 can generate O_2_^−•^ under US irradiation using DHR123 as a O_2_^−•^ probe, which can specific detection of O_2_^−•^. No significant change of the fluorescence intensity of DHR123 was observed in IR783 under US irradiation (Figure S5, Supporting Information), indicating that IR783 can only produce ^1^O_2_ under US irradiation. When the ratio of IR783 to Cu^2+^ was 1:1, the decline of DPBF peak in Cu‐IR783 slowed down under US irradiation compared with the free IR783 (Figure 2g), suggesting that the sonodynamic performance of IR783 was passivated by assembling to Cu‐IR783 NPs. The completely passivated sonodynamic activity of IR783 was achieved at the IR783/Cu^2+^ ratio of 1: 4 (Figure 2h), revealing negligible absorption change of Cu‐IR783 after the treatment of US irradiation. Almost no ^1^O_2_ generation can be detected in Cu‐IR783 at the IR783/Cu^2+^ ratio of 1: 4, which exhibited that the rate constant of ^1^O_2_ generation was as low as 0.01 min^−1^ (Figure 2i). These results forcefully demonstrated that the NIR imaging ability and sonosensitization function of IR783 in the nanoassemblies were completely passivated under the normal physiological conditions.

Considering that GSH could act as a hole sacrificial agent to inhibit electron‐hole pair recombination, we then investigated the GSH depletion capability of IR783 in the presence of US treatment. Figure 2j exhibited that the characteristic absorption peak of DTNB, which acted as the specific probe to detect GSH, was significantly decreased after US irradiation for 10 min in the presence of IR783, manifesting the efficient GSH depletion ability of the free IR783. However, the GSH consumption capability of IR783 was decreased after the addition of Cu^2+^ (Figure 2k), which revealed that the GSH consumption rate was calculated to be ≈52% at the IR783/Cu^2+^ ratio of 1: 1 (Figure S6, Supporting Information). Notably, almost no GSH consumption can be detected in Cu‐IR783 NPs after US irradiation for 10 min at the IR783/Cu^2+^ ratio of 1: 4 (Figure 2l), demonstrating that the GSH depletion ability of IR783 was completely passivated by assembling to Cu‐IR783 NPs.

### 2.3 pH‐Responsive Disassembly of Cu‐IR783 NPs

**Figure 3:**
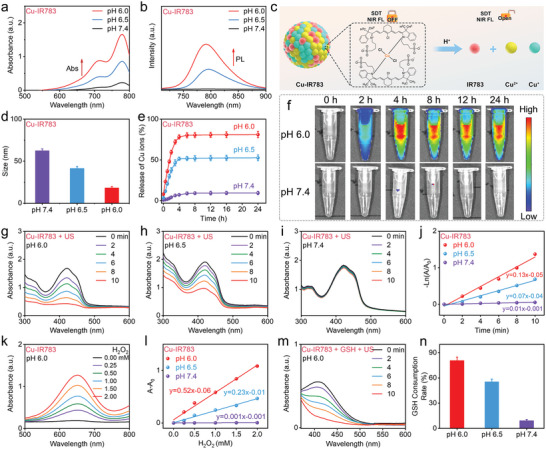
pH‐Responsive Disassembly of Cu‐IR783 NPs. a,b) Absorption spectra (a) and NIR fluorescence spectra (b) of nanoassemblies at varied pH (6.0, 6.5, and 7.4). c) Schematic illustration of the disassembly of nanoassemblies for the activation of sonodynamic activity and NIR imaging ability of IR783. d) Hydrodynamic diameter of nanoassemblies at varied pH (6.0, 6.5, and 7.4). e) The release rate of Cu^2+^ from nanoassemblies at varied pH (6.0, 6.5, and 7.4). f) NIR images of nanoassemblies after incubation of different times at varied pH (6.0 and 7.4). g–i) Absorption spectrum of nanoassemblies under US irradiation at varied pH (6.0, 6.5, and 7.4). j) Comparison of the sonodynamic activity of nanoassemblies at varied pH (6.0, 6.5, and 7.4). k) Absorption spectra of nanoassemblies at pH 6.0 in the presence of H_2_O_2_ with varied concentrations. l) Comparison of the chemodynamic activity of nanoassemblies at varied pH (6.0, 6.5, and 7.4). m) Absorption spectra of nanoassemblies in the presence of GSH and US irradiation at pH 6.0. n) Comparison of the GSH depletion ability of nanoassemblies at varied pH (6.0, 6.5, and 7.4). Data are presented as the mean ± SD. (n = 3).

After confirming the passivation of sonodynamic and NIR imaging functions of IR783, we then investigated the responses of nanoassemblies toward internal stimuli of TME, which was characterized by weak acidity and overexpressed GSH. Considering that the completely passivated sonodynamic activity and completely quenched fluorescence of IR783 was detected at the IR783/Cu^2+^ ratio of 1: 4, the type of Cu‐IR783 used for the experiments after section 2.3 is Cu‐IR783 (IR783: Cu^2+^ = 1: 4). As presented in **Figure** **3**a, the absorption spectrum of Cu‐IR783 was gradually increased as the pH value decreasing. The characteristic absorption peaks of IR783 located at ≈700 and 780 nm were almost complete recovery in Cu‐IR783 NPs at pH 6.0, while no significant peaks of IR783 can be detected in Cu‐IR783 NPs at pH 7.4, demonstrating that the release of IR783 only occurred in acidic condition. In addition to absorption spectrum, we also measured the NIR fluorescence spectrum of Cu‐IR783 NPs at varied pH. Figure 3b exhibited that the NIR emission of IR783 from Cu‐IR783 NPs was significantly increased with decreasing the pH value. The NIR fluorescence recovery phenomenon was also observed in Figure 3f, which revealed that the NIR fluorescence of Cu‐IR783 NPs was completely recovered after incubation of 4 h at pH 6.0. In addition, the fluorescence recovery of Cu‐IR783 NPs at pH 6.5 was lower than that at pH 6.0 (Figure S7, Supporting Information), suggesting the insufficient IR783 release from Cu‐IR783 NPs at pH 6.5. Notably, no significant NIR fluorescence signal can be detected in Cu‐IR783 NPs after incubation of 24 h at pH 7.4, demonstrating that the disassembly of nanoassemblies cannot occur at the normal physiological conditions. The disassembly of nanoassemblies was then evaluated by measuring their hydrodynamic diameter at different pH. As illustrated in Figure 3d, the decreased particle size of Cu‐IR783 NPs was detected at pH 6.5 and 6.0, while the hydrodynamic diameter of nanoassemblies exhibited insignificant change after incubation of 24 h at pH 7.4. We also utilized ICP‐OES to measure the concentrations of released Cu ions. Figure 3e exhibited that the release population of Cu^2+^ and Cu^+^ in Cu‐IR783 NPs was determined to be ≈80% after incubation of 4 h at pH 6.0, which was much higher than that at pH 6.5 and 7.4, clearly confirming the successful disassembly of nanoassemblies. We also performed the XPS spectrum of Cu‐IR783 after the release under acidic conditions. As depicted in Figure S8 (Supporting Information), the ratio of Cu^+^ to Cu^2+^ was determined to be ≈2.0, which was similar to that of Cu‐IR783 before the release under acidic conditions (Figure 1h). The decreased Zeta potential of nanoassemblies after incubation at pH 6.0 was detected compared with that at pH 7.4 (Figure S9, Supporting Information). These above results demonstrated that Cu‐IR783 NPs could response to the TME with weak acidity feature and release IR783 and Cu ions.

Having confirmed the disassembly and NIR fluorescence recovery of Cu‐IR783 NPs in acidic condition, we then investigated whether the sonodynamic activity of IR783 could be activated with the disassembly of Cu‐IR783 NPs, and whether this process would trigger other functions after Cu^2+^ and Cu^+^ release. As depicted in Figure 3g–i, the activated sonodynamic activity of IR783 was detected in Cu‐IR783 NPs after incubation at pH 6.0, while no ROS generation can be detected in Cu‐IR783 NPs after incubation at pH 7.4, demonstrating that the sonodynamic activity of IR783 only can be recovered in acidic condition. The rate constant of ^1^O_2_ generation in Cu‐IR783 NPs at pH 6.0 was determined to be 0.13 min^−1^ (Figure 3j), suggesting the efficient recovery of the sonodynamic activity of IR783. The chemodynamic activity of Cu‐IR783 NPs after disassembly was then investigated at varied pH. As illustrated in Figure 3k,l, the generation of •OH was detected in Cu‐IR783 NPs after incubation at pH 6.0. In addition, the free IR783 exhibited insignificant ROS production after incubation at pH 6.0 (Figure S10, Supporting Information), elucidating that •OH was generated by Cu^+^‐mediated Fenton‐like reaction after disassembly of Cu‐IR783 NPs. Moreover, no •OH production can be detected in Cu‐IR783 NPs after incubation at pH 7.4 (Figure S11, Supporting Information), demonstrating that Cu^+^ cannot be released from Cu‐IR783 NPs at the normal physiological conditions, which was favorable for avoiding the side effects of Cu ions on the normal tissues. The GSH depletion ability of Cu‐IR783 NPs under US irradiation was also recovered after incubation at pH 6.0 (Figure 3m,n). However, GSH cannot be consumed by Cu‐IR783 NPs under US irradiation after incubation at pH 7.4 (Figure S12, Supporting Information), which could be ascribed to the insignificant release of Cu^2+^ from Cu‐IR783 NPs. Hence, the acidic pH in the TME can trigger multiple responses of Cu‐IR783 NPs, including NIR fluorescence recovery, sonodynamic activation, and Cu^+^‐mediated OH generation, manifesting the significant potentials of Cu‐IR783 NPs as an intelligent sonosensitizer or nanozyme for the visualized in‐situ SDT and CDT.

### 2.4 In Vitro Tumor‐Cell‐Specific Activation of Cu‐IR783 NPs for SDT/CDT

**Figure 4:**
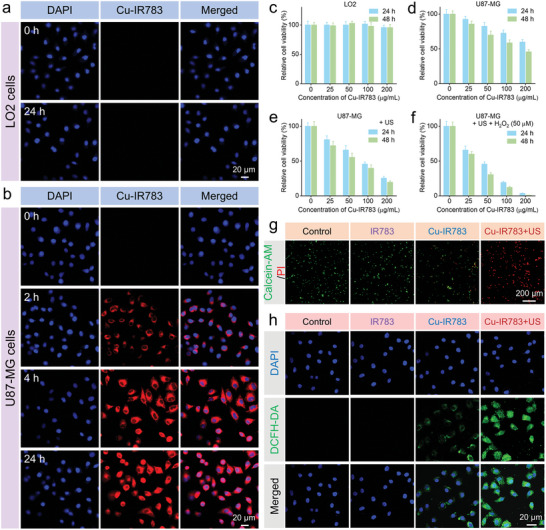
In Vitro Tumor‐Cell‐Specific Activation of Cu‐IR783 NPs for SDT/CDT. a,b) Confocal images of LO2 (a) and U87‐MG (b) cells incubated with nanoassemblies for different times. c–f) Cytotoxicity of nanoassemblies against LO2 (c) and U87‐MG (d–f) with or without US irradiation. g,h) Live/dead and ROS staining of U87‐MG cells after different treatments. Data are presented as the mean ± SD. (n = 5).

The pH‐responsive disassembly behaviors of nanoassemblies were then assessed at the cellular level. After incubation of Cu‐IR783 NPs with the normal human liver LO2 cells for 24 h, no significant red fluorescence signal can be detected in the LO2 cell (**Figure** **4**a; Figure S13a, Supporting Information), suggesting that IR783 cannot be released from Cu‐IR783 NPs in the normal cells. In contrast, the weak IR783 signal was observed in the U87‐MG cells after treating with Cu‐IR783 NPs for 2 h (Figure 4b; Figure S13b, Supporting Information), indicating that IR783 was released from Cu‐IR783 NPs after 2 h‐incubation. After the incubation time prolonged to 4 h, a large number of red fluorescence represented IR783 can be found in the U87‐MG. The fluorescence intensity of IR783 in U87‐MG after incubation of 4 and 24 h was similar, which illustrated that IR783 was completely released from Cu‐IR783 NPs after incubation of 4 h. The release of IR783 was also verified by the flow cytometry (Figure S14, Supporting Information), which revealed the strong IR783 signal after incubation of 4 h. These results not only revealed that IR783 can be released from Cu‐IR783 NPs in tumor cells and restore its NIR fluorescence imaging ability, but also demonstrated that the tumor‐cell‐specific release of IR783 would avoid the obvious toxic effects to the normal tissues and organs.

Having demonstrated the tumor‐cell‐specific disassembly of nanoassemblies, we then evaluated whether Cu‐IR783 would induce cytotoxicity to the normal cells. As shown in Figure 4c, no significant cytotoxicity of nanoassemblies against LO2 cells was detected at the concentration of 200 µg mL^−1^. The good biocompatibility of the free IR783 was also demonstrated (Figure S15, Supporting Information). In contrast, the cell viability of U87‐MG cells treated with Cu‐IR783 NPs at the same concentration was decreased to ≈45% (Figure 4d), demonstrating the tumor‐cell‐specific cytotoxicity of nanoassemblies. The cytotoxicity effect of nanoassemblies on the cancer cells was further enhanced in the presence of US irradiation (Figure 4e), elucidating that IR783 could be released from Cu‐IR783 NPs to exert sonodynamic activity. To further amplify the chemodynamic activity of Cu^+^, the additional H_2_O_2_ was introduced to the MTT assay. The higher cytotoxicity of U87‐MG cells was detected in the Cu‐IR783 + H_2_O_2_ group compared with the Cu‐IR783 alone group (Figure S16, Supporting Information), demonstrating that the Cu‐IR783 NPs can induce stronger CDT effect in the presence of additional H_2_O_2_. Figure 4f exhibited that almost all U87‐MG cells were dead after treating with Cu‐IR783 NPs + US, indicating the synergistic SDT and CDT via Cu‐IR783 NPs after disassembly to IR783 and Cu ions. The similar antitumor effectiveness of Cu‐IR783‐mediated SDT, CDT, and cuproptosis was also detected in another tumor cell lines including A549 and Hela cells (Figure S17, Supporting Information).

In addition to MTT assay, we also performed live/dead cell staining to investigate the therapeutic effects of in‐situ SDT and CDT via Cu‐IR783 NPs. As illustrated in Figure 4g and Figure S18 (Supporting Information), the strong green fluorescence signal was detected in the control, US alone, and IR783 alone groups. A weak red fluorescence signal was observed in the Cu‐IR783 alone group, suggesting that Cu^+^ can be released to mediate Fenton‐like reaction. In addition, the more dead cells were detected in the Cu‐IR783 + H_2_O_2_ group compared with the Cu‐IR783 alone group, confirming that the Cu‐IR783 NPs can induce stronger CDT effect in the presence of additional H_2_O_2_. After treatment with Cu‐IR783 and US irradiation, no notable green fluorescence was observed in U87‐MG cells, demonstrating the efficient therapeutic effects of in‐situ SDT and CDT via Cu‐IR783 NPs.

We also carried out ROS staining to investigate the mechanism of synergistic SDT and CDT via Cu‐IR783 NPs. Figure 4h and Figure S19 (Supporting Information) exhibited that no ROS signal was detected in the control, IR783 alone, and US alone groups. The stronger green fluorescence signal was observed in the Cu‐IR783 + H_2_O_2_ group compared with the Cu‐IR783 alone group, indicating that more ROS can be produced by Cu‐IR783 in the presence of additional H_2_O_2_. In addition, the strongest green fluorescence can be detected in the Cu‐IR783 + US group compared with the other groups, manifesting that the abundant ROS can be induced by Cu‐IR783‐mediated in‐situ SDT and CDT after disassembly. The ROS‐induced apoptosis was then demonstrated by flow cytometry (Figure S20, Supporting Information), which revealed that ≈90% U87‐MG cells were apoptosis after treating with Cu‐IR783 under US irradiation. These results demonstrated the efficient therapeutic effects of in‐situ SDT and CDT via Cu‐IR783 NPs.

### 2.5 In Vitro Anticancer Mechanism of Cu‐IR783 NPs

**Figure 5:**
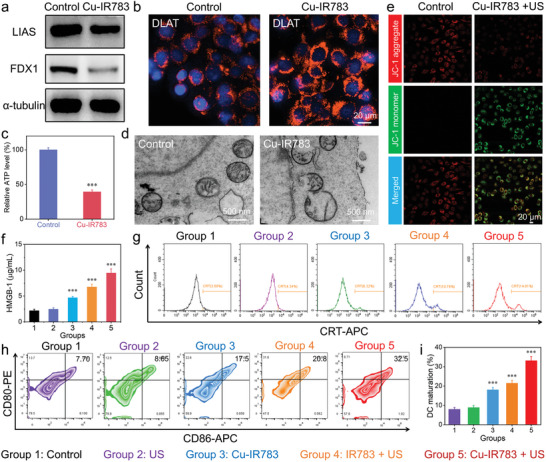
In Vitro Anticancer Mechanism of Cu‐IR783 NPs. a) LIAS and FDX1 protein expression in 4T1 cells after different treatments. b) DLAT fluorescence images of 4T1 cells after different treatments. c) Intracellular ATP levels of 4T1 cells after different treatments. d) Bio‐TEM images of 4T1 cells before and after Cu‐IR783 treatment. e) Confocal images of 4T1 cells stained with JC‐1 after different treatments. f,g) HMGB1 and CRT levels in 4T1 cells after different treatments. h,i) The expression of CD80 and CD86 in DCs after different treatments determined by flow cytometry. Statistical significance between the experimental group and the control group is calculated with a two‐tailed Student's *t*‐test. Data are presented as the mean ± SD. (n = 3). ^***^ *p* < 0.001.

Considering the presence of Cu ions and the pH‐responsive release behaviors of Cu‐IR783 NPs, we then evaluated the effects of Cu‐IR783 NPs on cuproptosis of cancer cells at the cellular level. It is well known that cuproptosis is sensitive to cancer cells with strong mitochondrial respiration ability, such as 4T1 cells.^[^ ^38^ ^]^ We thus selected 4T1 cells as the investigation object to evaluate the influence of Cu‐IR783 NPs on the cuproptosis pathway. Western blot (WB) analysis was initially performed to determine the expression of the cuproptosis‐associated protein in 4T1 cells after treating with Cu‐IR783. The interaction between Cu ions and dihydrolipoamide S‐acetyltransferase (DLAT) could trigger the aggregation or oligomerization of DLAT, which is a distinctive hallmark of cuproptosis.^[^ ^43^ ^]^ As depicted in **Figure** **5**b, the obvious aggregation of DLAT was detected in 4T1 cells after treating with Cu‐IR783, suggesting that cuproptosis was successfully induced by Cu‐IR783 after disassembly. In addition to DLAT oligomerization, we also investigated other representative features of cuproptosis. Figure 5a exhibited that the expression of lipoyl synthase (LIAS) and ferredoxin 1 (FDX1) was decreased in 4T1 cells after incubation of Cu‐IR783, indicating that the loss of iron‐sulfur proteins was induced by Cu‐IR783‐mediated cuproptosis. We then evaluated the ATP level in 4T1 cells after treating with Cu‐IR783. The significantly decreased ATP level in 4T1 cells can be observed in the Cu‐IR783 group (Figure 5c), demonstrating that Cu‐IR783 could inhibit ATP production owing to the mitochondrial damage. The mitochondrial damage caused by Cu‐IR783‐mediated cuproptosis was further investigated by the bio‐TEM characterization. As presented in Figure 5d, the obvious mitochondrial damage can be detected in 4T1 cells after incubation of Cu‐IR783. To further confirm the mitochondrial damage, we also measured the membrane potential change of mitochondria in 4T1 cells after different treatments using JC‐1 as the probe. The strong green fluorescence was detected in 4T1 cells after treating with Cu‐IR783 (Figure S21, Supporting Information), suggesting the dysfunctional mitochondria induced by Cu‐IR783. In addition, the Cu‐IR783 + US group exhibited the strongest green fluorescence signal, suggesting that Cu‐IR783‐mediated cuproptosis would amplify the therapeutic effects of SDT and CDT to induce severe mitochondrial dysfunction. These results demonstrated that Cu‐IR783 NPs could induce cuproptosis to enhance the SDT/CDT‐induced apoptosis for achieving the efficient cell death.

After demonstrating the Cu‐IR783‐induced apoptosis and cuproptosis, we further explored the cell death mechanism associated with the combined CDT and SDT. Many groups have reported that ROS‐induced ICD could lead to the exposure of three types of DAMPs, such as calreticulin (CRT) and high mobility group box 1 (HMGB1).^[^ ^62^, ^63^, ^64^ ^]^ The engulfment of apoptotic cells can be promoted by the exposed CRT, which would bind to the CD91 receptor on the DC membrane.^[^ ^62^, ^65^ ^]^ The semi‐quantitatively analyses were thus performed to measure the expression of CRT on the plasma membrane of 4T1 cells through flow cytometry after different treatments. Figure 5g and Figure S22 (Supporting Information) revealed that the strongest CRT signal was detected in the Cu‐IR783 + US group, elucidating that the most ROS were generated by Cu‐IR783 under US irradiation after the combination therapy of SDT, CDT, and cuproptosis. HMGB1 is categorized as a secondary key DAMP, acting as an attractant for immune cells by binding to antigen‐presenting cells (APC) and inducing the secretion of pro‐inflammatory cytokines.^[^ ^66^, ^67^ ^]^ This process serves to promote the activation of protective immunity when HMGB1 is released into the extracellular matrix.^[^ ^65^, ^66^ ^]^ Based on this situation, the ELISA assay was carried out to assess the HMGB1 level in 4T1 cells after different treatments. As depicted in Figure 5f, the Cu‐IR783 + US group exhibited the highest HMGB1 level compared with the other groups, implying that the synergistic SDT, CDT, and cuproptosis via Cu‐IR783 NPs could induce the strongest cell damage. These results clearly demonstrated that the strong ICD effects could be induced by producing abundant ROS and activating cuproptosis.

Given the potential of DAMPs released through Cu‐IR783‐induced ICD to bind to DCs and trigger adaptive immune responses, we proceeded to assess the percentage of mature DCs after different treatments through flow cytometry analysis. Upon isolating immature DCs from bone marrow‐derived cells and incubating with IL‐4 and GM‐CSF, we evaluated the effect of Cu‐IR783 NPs on the maturation of DCs through determining the expression of CD86 and CD80 on DCs after adding the supernatant of 4T1 cells treated with Cu‐IR783 NPs. As presented in Figure 5h, the higher proportion of CD80^+^CD86^+^ DCs was detected in the Cu‐IR783 alone group compared with the control and US alone groups, demonstrating that Cu‐IR783‐mediated CDT could produce ROS to induce ICD for the promoting the maturation of DCs. In addition, the IR783 + US group exhibited the higher proportion of CD80^+^CD86^+^ DCs (Figure 5i), which could be attributed to the IR783‐mediated SDT. More importantly, the highest proportion of CD80^+^CD86^+^ DCs can be found in the Cu‐IR783 + US group, suggesting that the strong immune response could be induced by Cu‐IR783 through the synergistic effects of SDT, CDT, and cuproptosis.

### 2.6 Validating the Tumor‐Specific Release of IR783 through In Vivo NIR Imaging

**Figure 6:**
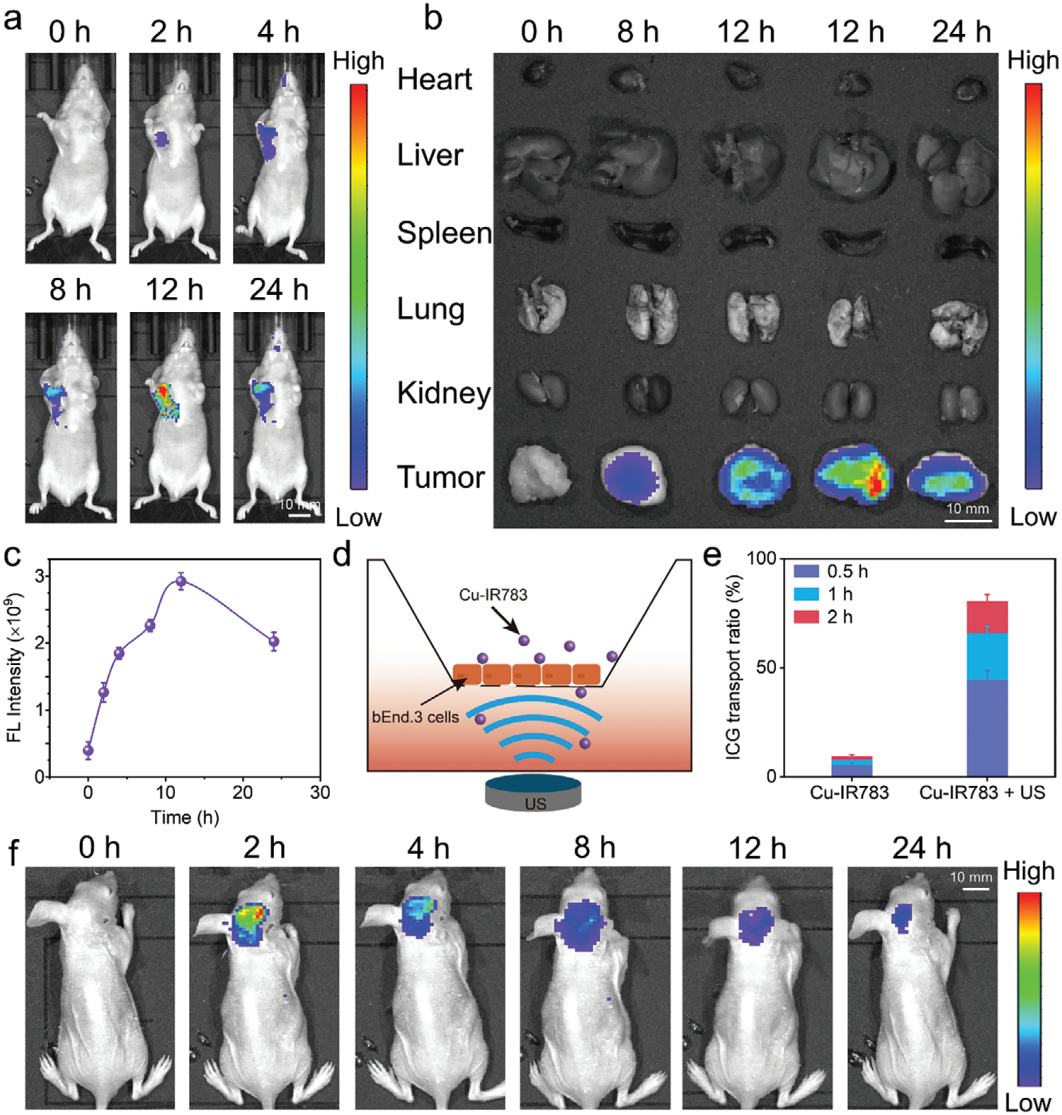
Validating the Tumor‐Specific Release of IR783 through In Vivo NIR Imaging. a–c) NIR imaging and the quantitative results of Cu‐IR783 NPs in a subcutaneous tumor model after intravenous injection. d) Schematic illustration of the evaluation of US‐assisted Cu‐IR783 transport across the in vitro BBB model using the Transwell assay. e) Measurements of the transport ratio of IR783 in the culture medium. f) NIR imaging of the orthotopic GBM‐bearing mice after administration of Cu‐IR783 NPs under US irradiation. Data are presented as the mean ± SD. (n = 3).

Next, we performed in vivo experiments to validate the tumor‐specific disassembly of Cu‐IR783 NPs and the TME‐responsive release of IR783. A subcutaneous tumor model was initially utilized to evaluate the in vivo NIR imaging of Cu‐IR783 NPs after intravenous injection. As shown in **Figure** **6**c, the NIR fluorescence signal in the tumor tissues was gradually increased after 2 h and reached maximum at 12 h, indicating that IR783 with NIR fluorescence imaging ability was released from Cu‐IR783 NPs owing to the response to TME. Moreover, no significant fluorescence signal was detected in the normal tissues or organs except for tumor tissues (Figure 6a), which confirmed that Cu‐IR783 NPs could only disassemble in the tumor tissues and thereby release IR783 to emit obvious NIR fluorescence signals. In contrast, mice administrated by the free IR783 exhibited evenly distributed NIR fluorescence signals (Figure S23, Supporting Information), suggesting that only a small amount of IR783 accumulated in tumors, while most of IR783 were evenly dispersed in normal tissues. We also utilized ex vivo imaging to verify the NIR fluorescence imaging results. Figure 6b and Figure S24 (Supporting Information) exhibited that the NIR fluorescence of IR783 was only detected in the tumor tissues after intravenous injection of nanoassemblies, demonstrating that the disassembly of nanoassemblies can only occur in the tumors owing to the stimulation of TME. These results forcefully demonstrated that the Cu^2+^‐induced IR783 assembly could effectively improve the signal‐to‐noise ratio to achieve the tumor‐specific NIR imaging, laying a good foundation for the subsequent realization of visualized SDT, CDT, and cuproptosis.

Having demonstrated the tumor‐specific NIR imaging ability of nanoassemblies, we then investigated the capability of Cu‐IR783 NPs to traverse the BBB with the aid of US irradiation. With a bEnd.3 cell monolayer cultured on Transwell, we constructed an in vitro simulated BBB model (Figure 6d). The higher transport ratio was observed in the Cu‐IR783 + US group compared with the Cu‐IR783 alone group (Figure 6e), illustrating that the US irradiation has the capacity to enhance the permeability of BBB for the promotion of Cu‐IR783 delivery. To further evaluate whether US could enhance the BBB penetration of Cu‐IR783 NPs in GBM, we established an orthotopic GBM model through orthotopic implantation with U87‐MG cells. The in vivo imaging system was utilized to measure the NIR fluorescence imaging of Cu‐IR783 NPs in GBM. As depicted in Figure 6f, the obvious NIR fluorescence signals were detected in the brain of mice after intravenous injection of nanoassemblies for 2 h under US irradiation, demonstrating that the permeability of the BBB and the accumulation of nanoassemblies were enhanced by the US treatment. The corresponding semiquantitative results of NIR fluorescence imaging can be found in Figure S25 (Supporting Information). Collectively, it can be concluded that Cu‐IR783 NPs could be effectively delivered to the orthotopic GBM with the help of US irradiation for the tumor‐specific NIR imaging.

### 2.7 In Vivo Antitumor Efficacy of Cu‐IR783 in Orthotopic GBM

**Figure 7:**
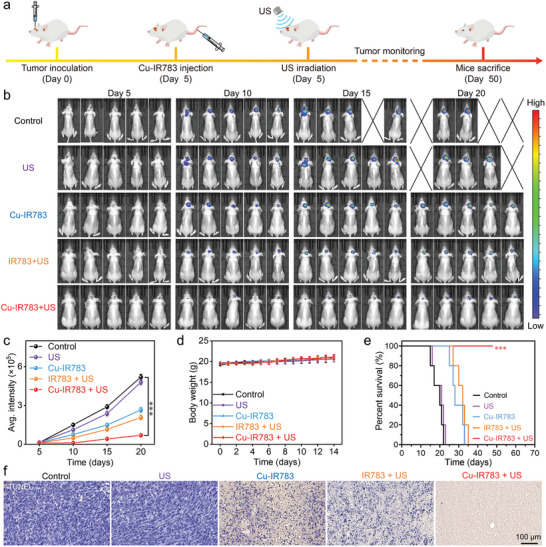
In Vivo Antitumor Efficacy of Cu‐IR783 in Orthotopic GBM. a) Schematic illustration of the treatments of orthotopic GBM through administration of Cu‐IR783 NPs with US assistance. b) Evaluation of the therapeutic effects through in vivo bioluminescence images after different treatments. c) The average bioluminescence signals in tumors after different treatments. d,e) Measurement of the body weight (d) and survival (e) of mice after different treatments. f) H&E staining of brain of mice after different treatments. Statistical significance between the experimental group and the control group is calculated with a two‐tailed Student's *t*‐test. Data are presented as the mean ± SD. (n = 5). ^***^ *p* < 0.001.

To investigate the antitumor efficacy of Cu‐IR783 NPs, an orthotopic GBM was established in mice and the administration of Cu‐IR783 NPs with US assistance was performed according to the tailored treatment plans (**Figure** **7**a). Following orthotopic U87‐MG GBM implantation, the mice were randomly divided into five groups including control, US, Cu‐IR783, IR783+ US, and Cu‐IR783 + US groups. Before intravenous injection of Cu‐IR783 NPs at day 5, the US irradiation was performed for 5 min to improve the penetration of Cu‐IR783 NPs into the GBM. According to the NIR fluorescence imaging results presented in Figure 6f, another US treatment was performed for 5 min at 2 h post‐injection. As presented in Figure 7b, the Cu‐IR783 + US group exhibited no detectable bioluminescence signal at day 10, whereas the other groups displayed prominent bioluminescence signals. During the treatment period, the bioluminescence signals of the control and US group showed a significant increase (Figure 7c), indicating the uncontrolled tumor growth. At day 20, the bioluminescence signals in the Cu‐IR783 + US group was much lower than that in the other groups, demonstrating that the efficient therapeutic effects of Cu‐IR783‐mediated synergistic SDT, CDT, and cuproptosis against brain tumors.

During the treatment period, the body weight of mice was monitored after different treatments to initially evaluate the biosecurity of Cu‐IR783‐mediated synergistic SDT, CDT, and cuproptosis. No significant fluctuation of mouse body weight in each group was observed in the curves in Figure 7d, implying the negligible side effects of these treatments in orthotopic GBM. In addition, the survival ratio of mice throughout the experiment was also monitored after different treatments. As presented in Figure 7e, all mice in the control group and US alone group died after 23 days, while the survival time of mice in Cu‐IR783 alone group and IR783+ US group extended to ≈30 days. Notably, all mice were survived after treating with Cu‐IR783 in the presence of US irradiation for 50 days, demonstrating the efficient antitumor efficacy of Cu‐IR783‐mediated synergistic SDT, CDT, and cuproptosis in treating in orthotopic GBM. The antitumor effectiveness of Cu‐IR783 + US was further demonstrated by the histopathological examination. TUNEL and H&E staining of brain sections in mice after administration for 20 days were depicted in Figure 7f and Figure S26 (Supporting Information). The significant cell apoptosis was detected in the Cu‐IR783 + US group, confirming the high‐efficiency therapeutic effects of Cu‐IR783 under US assistance. These results forcefully demonstrated that Cu‐IR783 NPs achieved satisfactory glioma‐targeting ability owing to the US‐enhanced permeability of the BBB and realized visualized in‐situ SDT, CDT, and cuproptosis due to the TME‐responsive disassembly of Cu‐IR783 to release IR783 and Cu ions.

### 2.8 In Vivo Antitumor Efficacy of Cu‐IR783 in Bilateral Tumor Model

**Figure 8:**
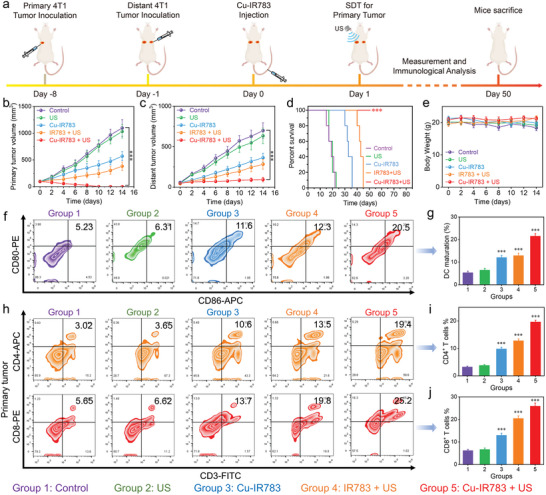
In Vivo Antitumor Efficacy of Cu‐IR783 in Bilateral Tumor Model. a) In vitro anticancer therapy of Cu‐IR783 is illustrated schematically, highlighting the synergetic SDT, CDT, and cuproptosis using a bilateral tumor model. b–e) Primary and distant tumor volume, survival, and body weight of mice after different treatments. f,g) Evaluation of the expression of CD80 and CD86 in the lymph nodes after different treatments. h–j) Evaluation of the expression of CD4^+^CD8^+^ T cells in the primary tumors after different treatments. Statistical significance between the experimental group and the control group is calculated with a two‐tailed Student's *t*‐test. Data are presented as the mean ± SD. (n = 5). ^***^ *p* < 0.001.

The leading cause of tumor‐related mortality in disease progression is distant metastasis.^[^ ^68^, ^69^, ^70^ ^]^ Hence, the optimal treatment strategy should encompass the eradication of both the primary tumor and metastatic tumor. Considering that the effectiveness of Cu‐IR783 NPs in the treatment of orthotopic GBM through the visualized in‐situ SDT, CDT and cuproptosis have been demonstrated, we further constructed bilateral tumor model using 4T1 cells to evaluate the therapeutic effects of Cu‐IR783 NPs through SDT/CDT‐induced immunotherapy (**Figure** **8**a). The reason for choosing 4T1 cells to construct a bilateral tumor model is that 4T1 cells not only have good tumorigenicity in Balb/c mice, but also have high invasiveness. As presented in Figure 8b,c, the treatment of Cu‐IR783 alone exhibited a certain therapeutic effect for the primary and distant tumors owing to the chemodynamic activity and cuproptosis effect of Cu‐IR783. For the IR783 + US group, the antitumor effectiveness was also detected, which could be ascribed to the sonodynamic activity of IR783 and ROS‐induced ICD effects. Notably, the strongest antitumor efficacy can be observed in the Cu‐IR783 + US group, revealing the complete eradication of primary tumor and the outstanding suppression effect on distant tumor, demonstrating that the treatment of Cu‐IR783 could induce robust immune response through the synergistic SDT, CDT, and cuproptosis. The therapeutic effects of Cu‐IR783‐mediated SDT, CDT, and cuproptosis were further assessed through histological analysis of the primary and distant tumors using TUNEL staining. As shown in Figure S27 (Supporting Information), the necrosis of the primary and distant tumors can be detected in the Cu‐IR783 + US group after the administration. Furthermore, the mice treated with Cu‐IR783 in the presence of US irradiation survived for more than 50 days (Figure 8d), suggesting that Cu‐IR783‐mediated SDT/CDT‐enhanced immunotherapy could significantly prolong the lifespan of mice. The aforementioned results forcefully indicated that the treatment of Cu‐IR783 plus US irradiation completely eradicated the primary tumors and effectively induced abscopal effects to inhibit the growth of the distant tumors.

We then investigated the immune responses induced by Cu‐IR783‐mediated SDT, CDT, and cuproptosis to elucidate the antitumor mechanism. By determining the CRT exposure level, HMGB1 release level, and intratumoral ATP levels, the in vivo ICD effects after different treatments were initially investigated. Figure S28 (Supporting Information) exhibited that the most CRT signals can be detected in the Cu‐IR783 + US group, showing the strongest green fluorescence signal compared with the other groups. Furthermore, the highest HMGB1 release was observed in the Cu‐IR783 + US group compared with the other groups (Figure S29, Supporting Information), elucidating that Cu‐IR783‐mediated SDT and CDT could induce the most pronounced ICD effect by generating more ROS. Apart from CRT and HMGB1, we also detected another biomarker of ICD effects through determining the ATP levels in each group. As presented in Figure S30 (Supporting Information), the Cu‐IR783 + US group showed the increased ATP level in the tumor tissues compared with the other groups, implying that Cu‐IR783‐induced antitumor immune responses could increase the intratumoral ATP level.

Having confirmed the in vivo ICD effects induced by Cu‐IR783 NPs, we then explored weather Cu‐IR783‐induced antitumor immune responses could stimulate the maturation of DCs. After preparing the single‐cell suspension of lymph nodes, we utilized the flow cytometry to assess the expression levels of CD80 and CD86 in each group. As depicted in Figure 8f, the treatment of PBS or US irradiation alone did not lead to a noticeable effect on the maturation of DCs, attributing to the insignificant ROS generation in the control group and US alone group. In contrast, an increase in the population of CD80^+^CD86^+^ matured DCs was detected in the Cu‐IR783 alone group and the IR783 + US group, demonstrating that a certain ROS can be produced by Cu‐IR783‐mediated CDT and IR783‐mediated SDT. Significantly, there was a notable increase in the populations of CD80^+^CD86^+^ matured DCs in the Cu‐IR783 + US group compared with the other groups (Figure 8g), suggesting the efficient antitumor immune responses of Cu‐IR783‐mediated SDT, CDT, and cuproptosis. We then explored whether T cell can be activated by Cu‐IR783 under US irradiation. After the administration of Cu‐IR783 in the presence of US irradiation, the higher proportion of CD4^+^CD8^+^ T cells in the primary tumors can be detected compared with the other groups (Figure 8h–j). In addition to the primary tumors, the increased proportion of CD4^+^CD8^+^ T cells after treating with Cu‐IR783 + US was also detected in the distant tumors (Figure S31, Supporting Information), clearly confirming that the synergistic SDT, CDT, and cuproptosis via Cu‐IR783 NPs could induce the robust immune responses to realize the enhanced immunotherapy. Given the significance of immune cytokine levels in DC maturation and T‐cell activation, an ELISA assay was carried out to evaluate the serum levels of cytokines after different treatments, such as tumor necrosis factor𝛼 (TNF‐𝛼), interferon‐𝛾 (IFN‐𝛾), and interleukin‐6 (IL‐6). As revealed in Figure S32 (Supporting Information), the Cu‐IR783 + US group exhibited the highest levels of TNF‐𝛼, IFN‐𝛾, and IL‐6 compared with the other groups. These above findings forcefully confirmed that the tumor‐specific disassembled Cu‐IR783 NPs could achieve visualized in‐situ SDT, CDT, and cuproptosis for the activation of antitumor immune responses.

Ensuring the biosafety of nanomedicine is paramount in guaranteeing their clinical efficacy for tumor treatment.^[^ ^71^, ^72^ ^]^ Therefore, we then performed a series of experiments to demonstrate the excellent biosafety of Cu‐IR783‐mediated visualized in‐situ tumor therapy. We performed the biodistribution analysis of Cu‐IR783 at different time points to assess the metabolic pathway of Cu‐IR783. Figure S33 (Supporting Information) exhibited that Cu‐IR783 were primarily accumulated in liver and spleen due to the capture of reticuloendothelial system. After 7 days post‐injection, there is no discernible Cu signal present in these organs, suggesting that Cu‐IR783 was cleared from the mice via the liver and spleen. During the therapeutic process of in vivo suppression of primary and distant tumors, no significant weight loss of mice can be detected in each group (Figure 8e), suggesting an extremely low adverse effect during therapy. We also evaluated the potential systemic damage of Cu‐IR783 mediated tumor therapy through H&E staining of the major organs of mice after administration. Figure S34 (Supporting Information) exhibited that no apparent pathological abnormalities were observed in the major organs across all therapeutic schedules, indicating that Cu‐IR783 NPs would not disassemble in these organs to induce the Fenton‐like reaction, sonodynamic activity, and cuproptosis effects. Subsequently, the hepatic and renal function markers of mice after different treatments were assessed to demonstrate the good biosafety of Cu‐IR783 NPs. As depicted in Figure S35 (Supporting Information), all the markers of the blood biochemistry and blood routine examination in each group were within the normal concentration ranges, implying the intravenous injection of Cu‐IR783 in the presence of US irradiation would not induce any physical disorders of mice. To further demonstrate the biosecurity of Cu‐IR783 NPs with tumor‐specific disassembly behaviors, we performed the H&E staining of the normal brain tissues near GBM during the in vivo antitumor efficacy evaluation in orthotopic GBM. The histological slices of brain tissues exhibited the excellent structural integrity after treating with Cu‐IR783 + US (Figure S36, Supporting Information), suggesting that IR783 and Cu ions cannot be released from Cu‐IR783 NPs to exert SDT, CDT, and cuproptosis on the normal tissues.

## 3 Conclusion

In summary, an intelligent antitumor theranostic platform with passivated sonodynamic activity and NIR imaging ability is constructed by simple assembling of IR783 and Cu^2+^. Through a facile assembly process, the obtained Cu‐IR783 NPs can overcome the disadvantage that the systemically distributed IR783 cannot realize the in‐situ and visualized SDT, thereby enhancing the tumor accumulation owing to the EPR effect induced by the increased particle size. In addition to the enhanced tumor accumulation, the merits of Cu‐IR783 NPs for potential clinical translation can be summarized as follows. First, although Cu‐IR783 NPs will inevitably accumulate in normal tissues, IR783 and Cu ions are unable to exert NIR imaging, sonodynamic, chemodynamic, and cuproptosis functions because the disassembly of Cu‐IR783 NPs only occurs in tumor tissues. Second, due to the TME‐responsive release of Cu‐IR783 NPs, the NIR fluorescence imaging ability and sonodynamic activity of IR783 can be specifically activated in tumor tissues, realizing the visualized in‐situ SDT. Moreover, the tumor‐specific disassembly features of Cu‐IR783 NPs not only achieve the deep tumor penetration due to the smaller size of the released IR783 and Cu ions, but also avoid the obvious side effects on normal tissues. Third, the tumor‐specific released Cu ions could not only realize the cascade amplification of ROS production through Cu^+^‐mediated Fenton‐like reaction to induce CDT effects, but also induce tumor‐specific cuproptosis‐mediated cell death owing to the overload of Cu ions. Finally, the immunosuppressive TME can be reversed by the significantly amplified ROS level and high‐efficiency cuproptosis, which could trigger ICD effects to induce robust systemic immune responses. Collectively, this advanced antitumor theranostic platform offers valuable insights into the passivation and selectively activation of sonodynamic activity and NIR imaging ability of sonosensitizers and fluorescent probes, potentially promoting the clinical transformation of SDT.

## 4 Experimental Section

### Synthesis of Cu^2+^‐Induced IR783 Assembly (Cu‐IR783)

To fabricate Cu^2+^‐induced IR783 assembly (Cu‐IR783), the mass ratio of IR783 and Cu^2+^ were 4: 1, 1: 1, and 1: 4. In brief, 5 mL of IR783 (1 mg mL^−1^) was first mixed with 5 mL of CuCl_2_ solution at different concentration (0.25, 1, or 4 mg mL^−1^). Thereafter, the mixture was kept at room temperature for 24 h under gentle stirring. After washing by centrifugation for three times, the final product was collected through centrifugation.

### Characterization

TEM images of Cu‐IR783 NPs were acquired by a JEM‐2100F microscope. XRD measurements of Cu‐IR783 NPs were conducted by Rigaku 18 KW D/max‐2550. The valence of elements in Cu‐IR783 NPs were measured by XPS. UV‐vis‐NIR absorbance spectrum were recorded by Hitachi 3100. Fluorescence spectrum was measured by Agilent Cary 5000 spectrophotometer.

### Sonodynamic Performance Measurements

The absorption and fluorescence spectrum were utilized to determine the production of ROS in the presence of IR783 and Cu‐IR783 under US irradiation (50 kHz, 1.0 W cm^−2^). In brief, 60 µL DPBF was added to 3 mL of IR783 or Cu‐IR783 solution (200 µg mL^−1^). The mixture was irradiated by US and the absorption changes of DPBF at 418 nm were measured to determine the rate constant of ^1^O_2_. For the detection of O_2_^−•^, DHR123 was used as an O_2_^−•^ indicator. IR783 was mixed with DHR123 (10 mM) in DI water. The mixture solution was exposed to US irradiation for different times, and the emission spectra were detected immediately after each irradiation (E_x_: 500 nm).

### GSH Depletion Performance Measurements

IR783 or Cu‐IR783 was first mixed with 1 mM GSH in PBS solution. After US irradiation, 300 µL of mixture was added to 2700 µL PBS followed by adding 2 µL of DTNB (0.2 mm). The GSH depletion ability of IR783 or Cu‐IR783 were calculated by detecting the changes of the absorption peak of DTNB at 412 nm.

### TME‐Responsive Disassembly of Cu‐IR783 NPs

Cu‐IR783 NPs (200 µg mL^−1^) were dispersed in solutions at varied pH (6.0, 6.5, and 7.4). After incubation, the absorption and fluorescence spectra of the disassembled Cu‐IR783 NPs were measured. The released Cu ions were collected for ICP‐OES measurements to determine the weight of Cu ions.

### Chemodynamic Performance Measurements

20 µL of TMB was added to the disassembled Cu‐IR783 solution in the presence of H_2_O_2_ with various concentrations (0, 0.25, 0.50, 1.00, 1.50, and 2.00 mm), respectively. Then, we detected the change of TMB characteristic absorption peak at 652 nm.

### MTT Assay

LO2, U87‐MG, and bEnd.3 cells were purchased from Cell Bank of Type Culture Collection of Chinese Academy of Sciences (Shanghai, China). U87‐MG cells were incubated in 96‐well plates for 24 h. 100 µL of culture medium containing Cu‐IR783 was then added to the wells and incubated with U87‐MG for 4 h. The US (50 kHz, 1.0 W cm^−2^) treatment was then performed for 5 min and the U87‐MG cells were then incubated with Cu‐IR783 for another 24 or 48 h. After incubation, MTT assay was performed according to the provided protocol. The biocompatibility evaluation of IR783 and Cu‐IR783 NPs was also performed using LO2 cells.

### In Vitro ROS Detection and Live/Dead Cell Staining

For in vitro ROS detection, U87‐MG cells were first seeded in confocal dishes and incubated for 24 h. The cells were then incubated with samples and US irradiation was conducted for 5 min. All the images were acquired by confocal microscope (Olympus, Japan). For live/dead cell staining, we utilized the fluorescence microscope (Olympus, Japan) to obtain the fluorescence images under the excitation wavelength of 488 nm for calcein AM and 561 nm for PI.

### Western Blotting Assay

4T1 tumor cells seeded in a 6‐well plate were cultured and incubated with Cu‐IR783 NPs. The cells were then incubated for 24 h and the cell lysates were then collected. The cell lysates incubated with antibodies to LIAS (1:1000, abcam), FDX1 (1:1000, abcam) or αtubulin (1:1000, abcam) were analyzed and detected by a chemiluminescent imaging system.

### Apoptosis Assay

U87‐MG cells were plated on 12‐well plate and they were cultured for 1 day. After different treatments including control, US alone, IR783 alone, Cu‐IR783 alone, IR783 + US, and Cu‐IR783 + US, the U87‐MG cells were incubated for another 24 h. US treatments were carried out for 5 min under the condition of 50 kHz and 1.0 W cm^−2^. Then, U87‐MG cells were collected by centrifugation and detected by flow cytometer (Beckman, CytoFLEX LX) using an Annexin V‐FITC/PI assay kit (Dojindo, Japan) according to the manufacturer's protocol.

### In Vitro Evaluation of Matured DCs

BMDCs were derived from the humerus and tibia of Balb/c mice (6‐8 weeks old), and then incubated in the RPMI‐1640 medium for 7 days, which containing GM‐CSF (20 ng mL^−1^) and IL‐4 (10 ng mL^−1^). On the seventh days, we incubated BMDCs with a suspension of 4T1 cells treated with different groups. The cells were then incubated for another 24 h followed by collecting the suspended cells. Flow cytometry was used to analyze the BMDCs which stained with antibodies (FITC‐anti‐mouse CD11c, APC‐anti‐mouse CD86, PE‐anti‐mouse CD80) (Biolegend, USA).

### In Vitro Detection of ICD Biomarkers

For the detection of HMGB1, the 4T1 cells were treated with different groups, including control, US, Cu‐IR783, IR783 + US, and Cu‐IR783 + US. The concentration of IR783 and Cu‐IR783 was 200 µg mL^−1^. The collected supernatants were measured with HMGB1 Elisa Kit (Beyotime, China) according to the instruction.

For the detection of CRT, the 4T1 cells were treated with different groups. For flow cytometry, the collected cells were first immobilized with immunostaining fixative for 5 min and then blocked by immunol staining blocking buffer for 30 min. The cells were incubated with primary antibody (CRT, Abcam, USA) for 60 min. After washing with immunol staining wash buffer for three times, the cells were then incubated with secondary antibody (Alexa Fluor 647, Beyotime, China) for 30 min according to the recommended protocol. Finally, the CRT content in cells after different treatments was detected by the flow cytometry.

### Drug Transport across In Vitro BBB Model

To construct the in vitro BBB model, bEnd.3 cells were implanted on the 6.5 mm Transwell with a 0.4 µm pore size polycarbonate membrane insert (Corning) and cultured for several days. Cu‐IR783 NPs were added in the apical chamber. Then, the Transwell was treated with or without US irradiation (50 kHz and 1.0 W cm^−2^) and incubated for another 2 h. Cu‐IR783 transport ratio through the in vitro BBB model was obtained by analyzing IR783 concentration in the culture medium of the basolateral compartment at 0.5, 1, and 2 h.

### Tumor Model

5‐week‐old female Balb/c nude mice and Balb/c mice were purchased from SLAC Laboratory Animal (Shanghai, China). All animal experiments were carried out under the permission by Institutional Animal Care and Use Committee of Naval Medical University (IACUC‐2012226). To establish a bilateral tumor model, 1×10^6^ mouse breast cancer 4T1 cells were inoculated in the left and right axilla of Balb/c mice. To establish an orthotopic implantation GBM model, U87‐MG cells were implanted into the brain striatum of the Balb/c nude mice. First, total experimental equipment was cleaned with 75% alcohol and put in the biosafety cabinet with ultraviolet sterilization for 0.5 h. The anesthetized mice were immobilized on a stereotactic frame. A small slit was made in the scalp of the mice. Next, U87‐MG cells (2.5 × 10^5^ in 10 µL medium) were inoculated into the right striatum of mice by using a digital stereotaxic instrument (RWD Life Science, China). Set the zero points in the intersection of sagittal and coronal. The coordinates were moved to x (bright lateral) of 1.5 mm, y (bregma) of 1.0 mm, and z (depth) of 3.0 mm. The growth of intracranial tumor cells was observed via an in vivo bioluminescence/fluorescence imaging system (PerkinElmer, USA).

### In Vivo NIR Fluorescence Imaging

For the subcutaneous tumor, Cu‐IR783 NPs (2 mg kg^−1^) were intravenously injected into mice. After intravenous injection of different time, an IVIS Lumina III in vivo Imaging System (PerkinElmer, USA) was utilized to obtain the NIR fluorescence images under the excitation and collection wavelength of 700 and 790 nm, respectively.

For the orthotopic implantation GBM model, Cu‐IR783 NPs (2 mg kg^−1^) were intravenously injected into GBM‐bearing mice after being treated with US irradiation on the brains of mice. The fluorescence images were acquired at different time intervals by an IVIS Lumina III in vivo Imaging System (PerkinElmer, USA).

### In Vivo Antitumor Efficacy of Cu‐IR783 in Orthotopic GBM

Five days after the implantation of the orthotopic GBM model, the brains of mice were treated with US irradiation to open the tight junctions of BBB and increase the penetration of nanoparticles into the GBM. Immediately after the completion of the first US treatment, 200 µL of PBS, IR783, and Cu‐IR783 NPs were i.v. injected into mice. Two hours later, when the Cu‐IR783 NPs were exposed to the US irradiation to produce ROS. The weight and survival rates of mice were recorded. Mice were also subjected to bioluminescence imaging with the injection of luciferin. The bioluminescence intensity of mice was observed every 5 days to evaluate the tumor development.

### In Vivo Antitumor Efficacy of Cu‐IR783 in Bilateral Tumor Model

1×10^6^ mouse breast cancer 4T1 cells were inoculated in the left and right axilla of mice as a bilateral tumor model. When the primary tumor grows to 100 mm^3^, we divided mice into five groups, including PBS, US, Cu‐IR783, IR783 + US, and Cu‐IR783 + US. After intravenous injection of IR783 or Cu‐IR783 for 24 h, the US irradiation was performed. The dose of IR783 or Cu‐IR783 was 2.0 mg kg^−1^. The TUNEL staining were performed through collecting the tumors in different groups on the second day after being treated with US irradiation. After 7‐day treatment, the primary tumors, distant tumors, and lymph nodes were collected for the analysis of DCs and T cells. The collected tissues were made into single‐cell suspensions by enzymatic digestion, grinding, and filtering. According to the similar protocol described above, the prepared suspensions were incubated with different antibodies and analyzed by flow cytometry. The proinflammatory cytokines including IL‐6, TNF‐α, and IFN‐γ in serum were detected by Elisa kits.

### Biosafety Evaluation

For the evaluation of in vivo long‐term toxicity, the healthy Balb/c mice were selected for these experiments. We divided mice into five groups, including PBS, US, Cu‐IR783, IR783 + US, and Cu‐IR783 + US. After intravenous injection of IR783 or Cu‐IR783 for 24 h, the US irradiation was performed. The dose of IR783 or Cu‐IR783 was 2.0 mg kg^−1^. After 14‐day treatment, the major organs were collected for the H&E staining. The routine blood tests and histology examinations were also performed to assess the in vivo long‐term toxicity of these samples by collecting the blood from mice in each group. For metabolic pathway analysis, after i.v. injection of Cu‐IR783 for 1, 3, or 7 days, the main organs from these mice were collected to conduct ICP‐MS analysis.

### Statistical Analysis

All data are presented as mean ± standard deviation. All experiments were performed with at least three independent replicates. All data were reported as the mean ± standard deviation (SD). All statistical analyses were performed by Origin 2018 and Excel. Statistical significance between the two groups was calculated with a two‐tailed Student's *t*‐test. ^*^ denotes a statistical significance (^*^ *p* < 0.05, ^**^ *p* < 0.01, and ^***^ *p* < 0.001) between the experimental data of two groups.

## Conflict of Interest

The authors declare no conflict of interest.

## Supporting information



Supporting Information

## Data Availability

The data that support the findings of this study are available from the corresponding author upon reasonable request.
