# Supplementary material for: Tumor Microenvironment Activated Cu Crosslinked Near‐Infrared Sonosensitizers for Visualized Cuproptosis‐Enhanced Sonodynamic Cancer Immunotherapy
Source: Adv Sci (Weinh). 2024 Sep 27;11(43):2407196. doi: 10.1002/advs.202407196 (PMC11578373; doi:10.1002/advs.202407196)
Supplement: Supplementary file 1 — Supporting Information [file ADVS-11-2407196-s001.docx]

Supporting Information

**Tumor Microenvironment Activated Cu** **Crosslinked Near-Infrared Sonosensitizers for Visualized Cuproptosis-Enhanced Sonodynamic Cancer Immunotherapy**

Jinyan Hu, Lang Yan, Zhi Cao, Bijiang Geng, Xiqian Cao, Bing Liu*, Jiaming Guo*, Jiangbo Zhu*,

J. Hu, L. Yan, B. Geng, X. Cao, J. Zhu

Department of Health Toxicology, College of Naval Medicine, Naval Medical University, Shanghai 200433, China

E-mail: jiangbozhu1@smmu.edu.cn

Z. Cao

Department of Urology, Changhai Hospital, Naval Medical University, Shanghai 200433, China

B. Liu

Department of Urology, The Third Affiliated Hospital, Naval Medical University, Shanghai, 200433, China

E-mail: 13501616398@163.com

J. Guo

Department of Radiation Medicine, College of Naval Medicine, Naval Medical University, Shanghai 200433, China.

E-mail: smmuguojiaming@126.com


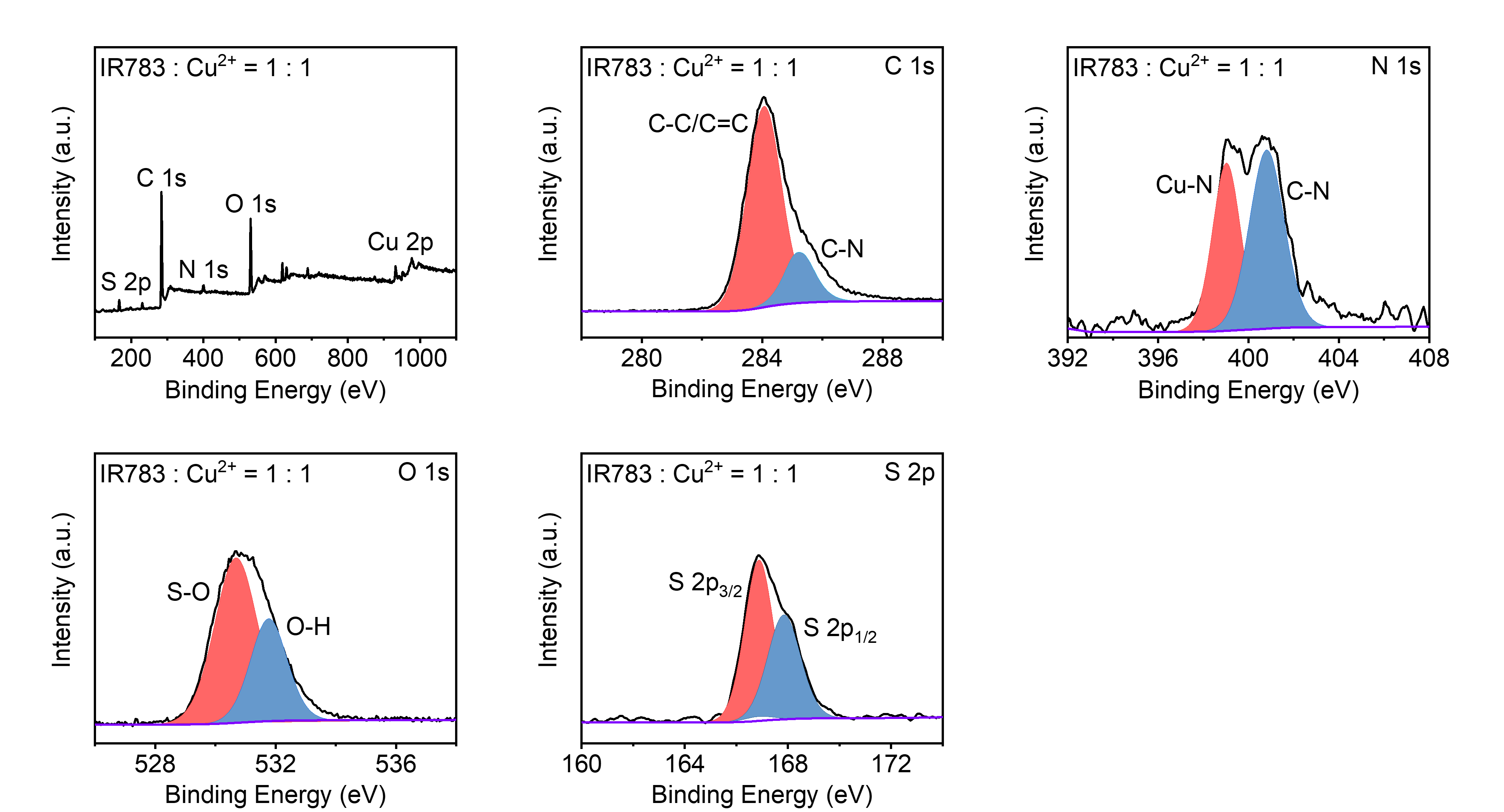


**Figure S1.** Survey XPS spectra, high-resolution C 1s, N 1s, O 1s, and S 2p spectra of Cu-IR783 NPs at IR783 to Cu^2+^ ratio of 1:1.


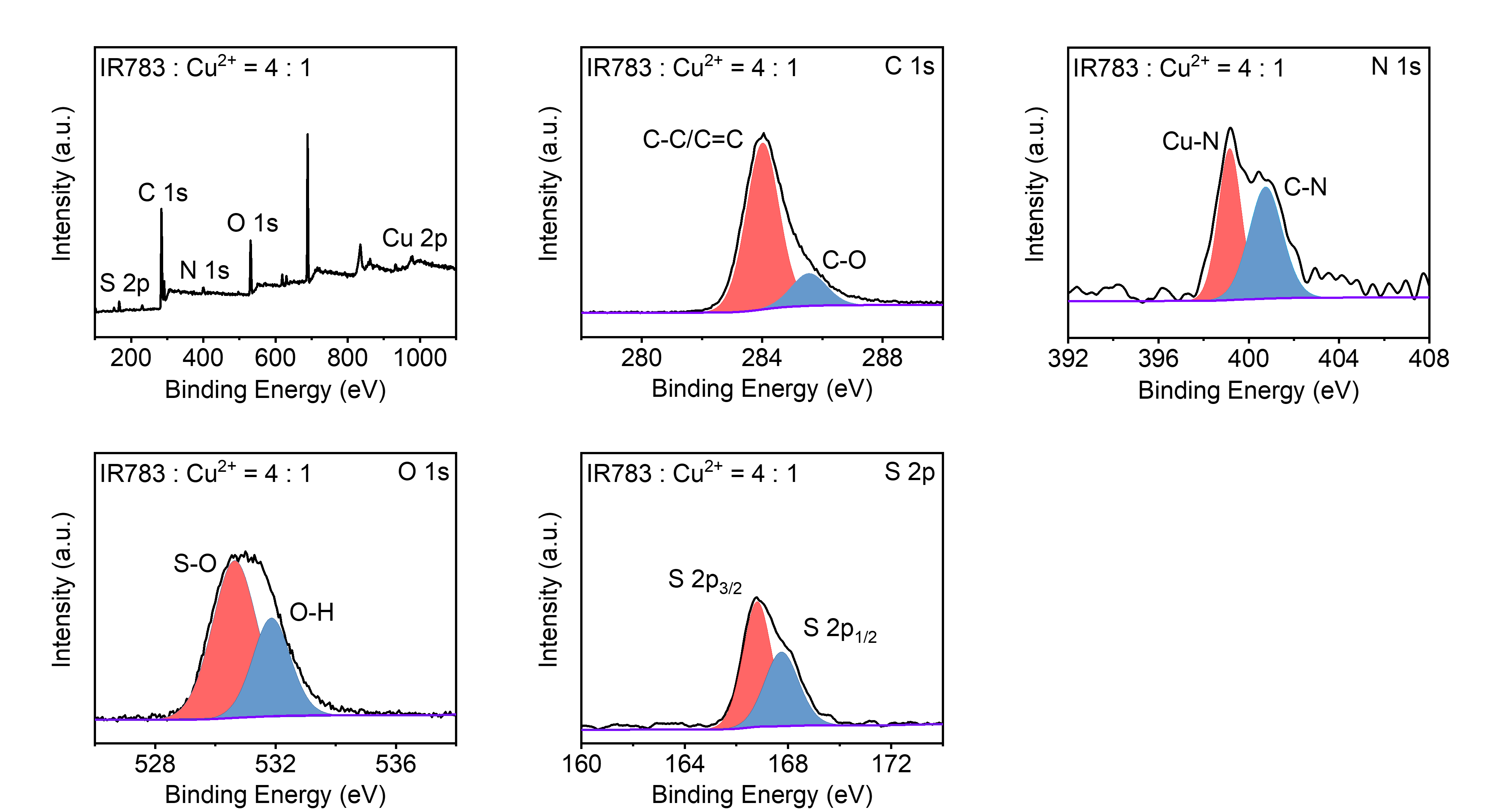


**Figure S2.** Survey XPS spectra, high-resolution C 1s, N 1s, O 1s, and S 2p spectra of Cu-IR783 NPs at IR783 to Cu^2+^ ratio of 4:1.


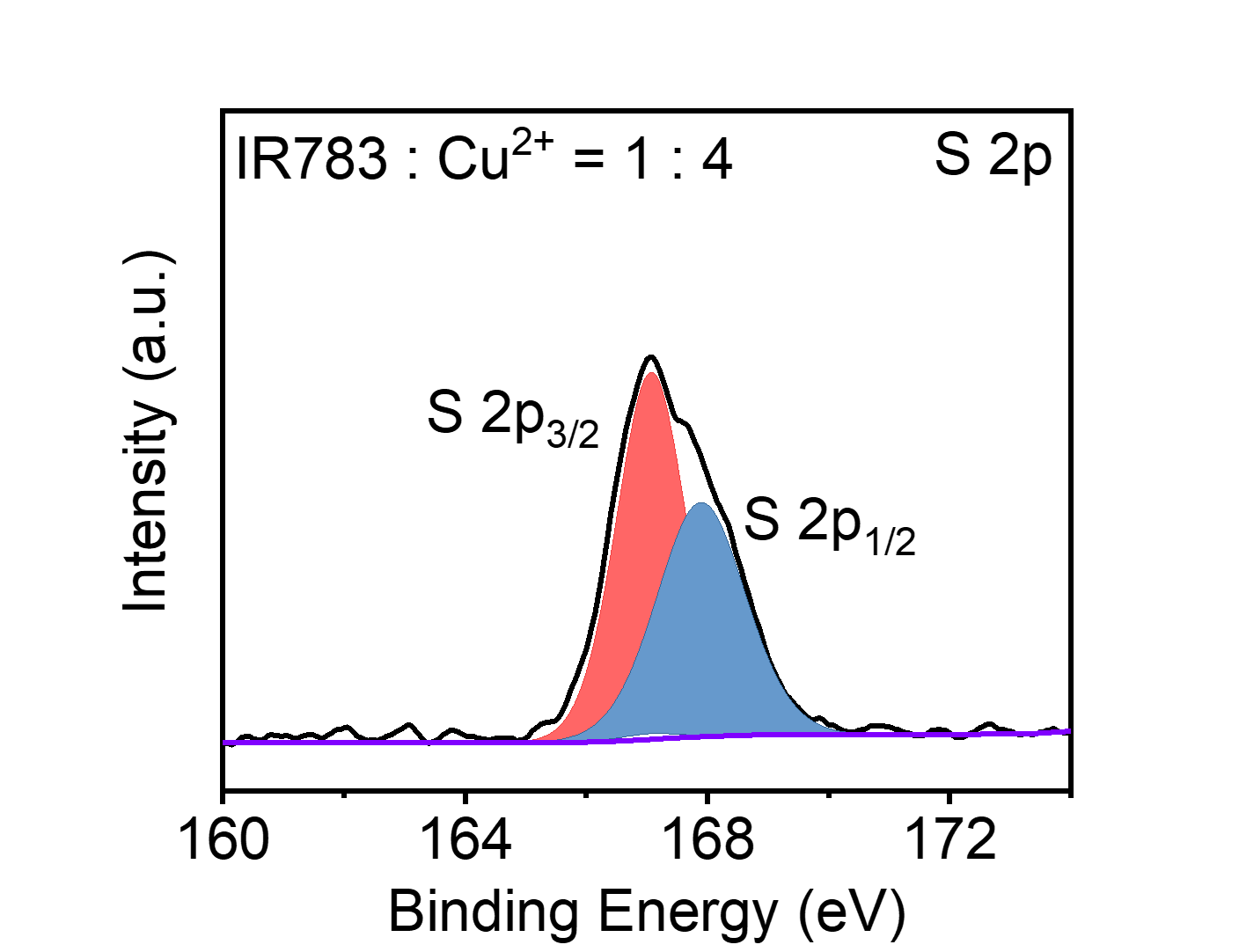


**Figure S3.** High-resolution S 2p spectrum of Cu-IR783 NPs at IR783 to Cu^2+^ ratio of 1:4.


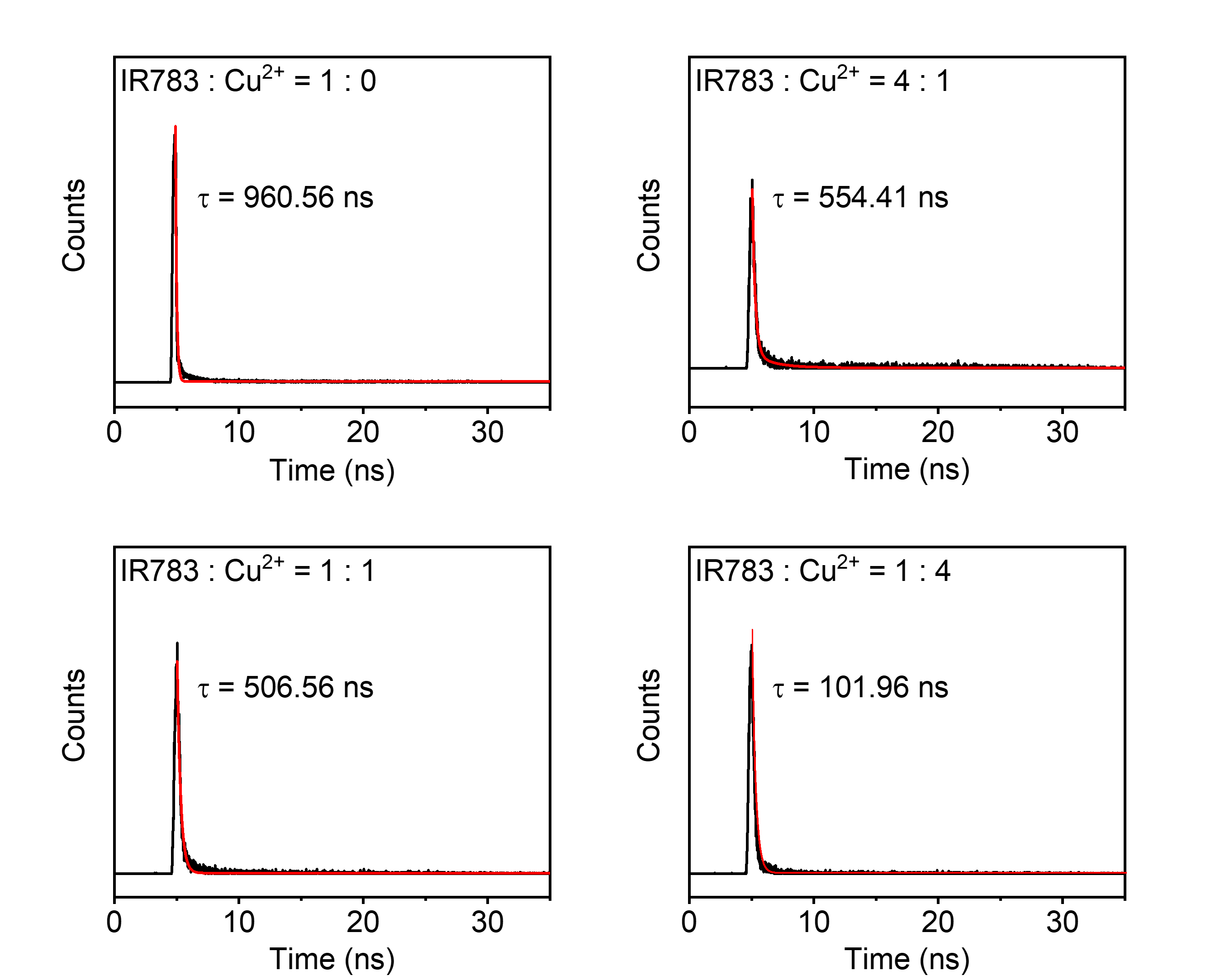


**Figure S4.** Time-resolved PL spectra and the corresponding fitting curves of Cu-IR783 NPs at different feeding concentrations of IR783 and Cu^2+^.


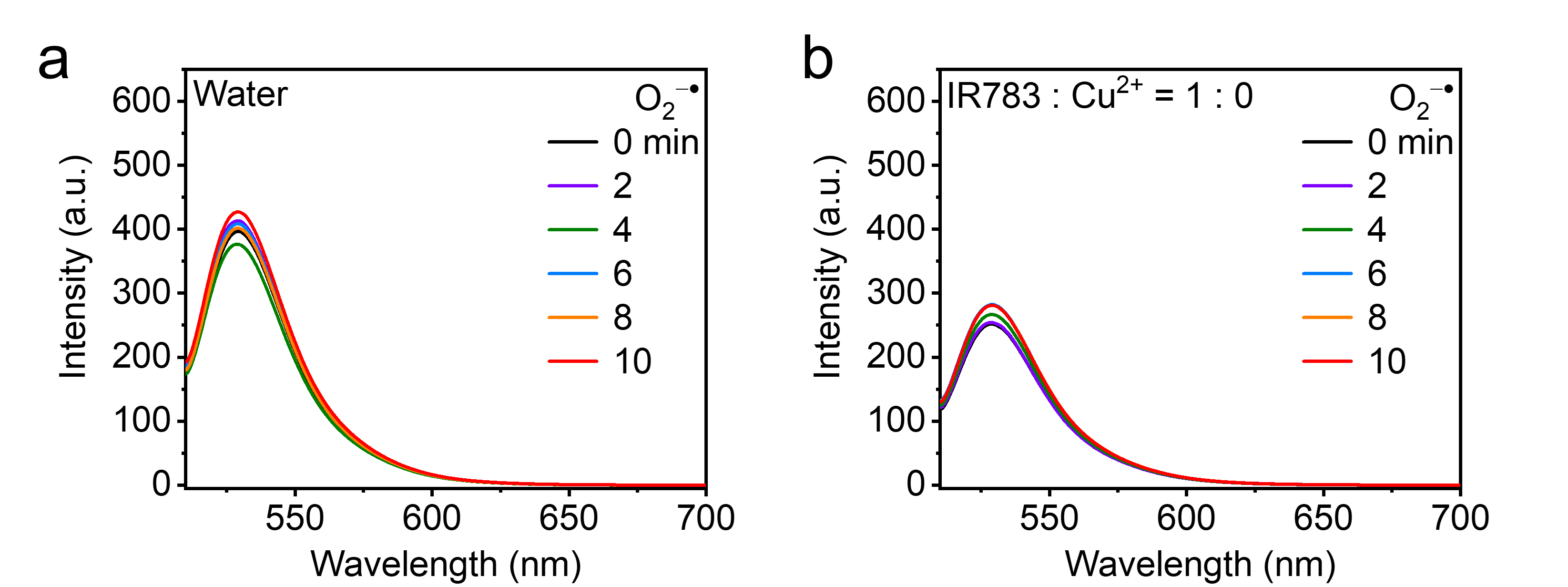


**Figure S5.** The fluorescence spectra of DHR123 in the presence of blank (a) and IR783 under US irradiation for different times (0-10 min).


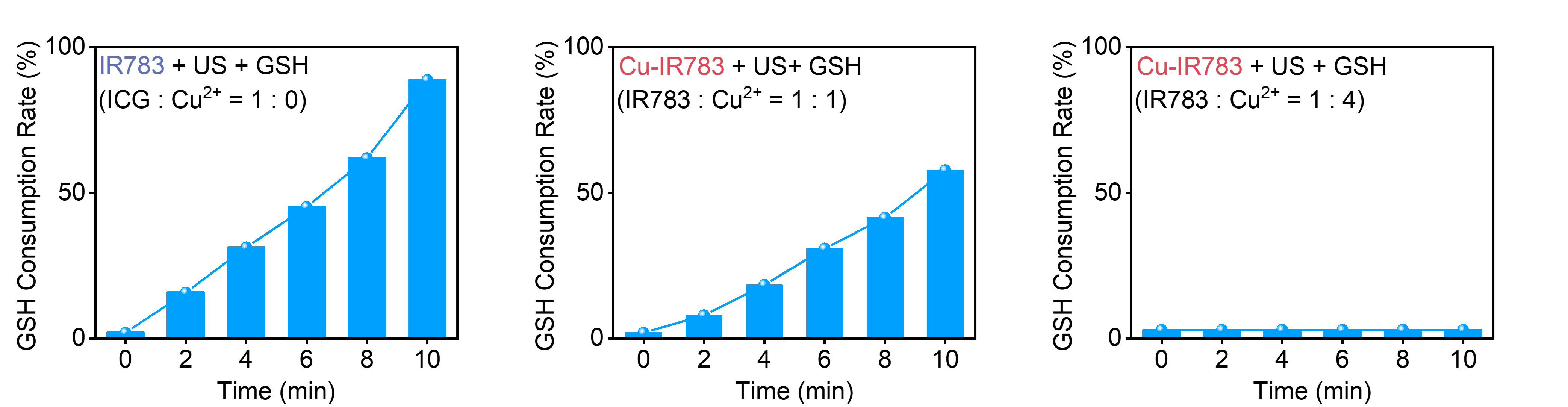


**Figure S6.** GSH depletion by holes produced from US-excited Cu-IR783 NPs at different feeding concentrations of IR783 and Cu^2+^.


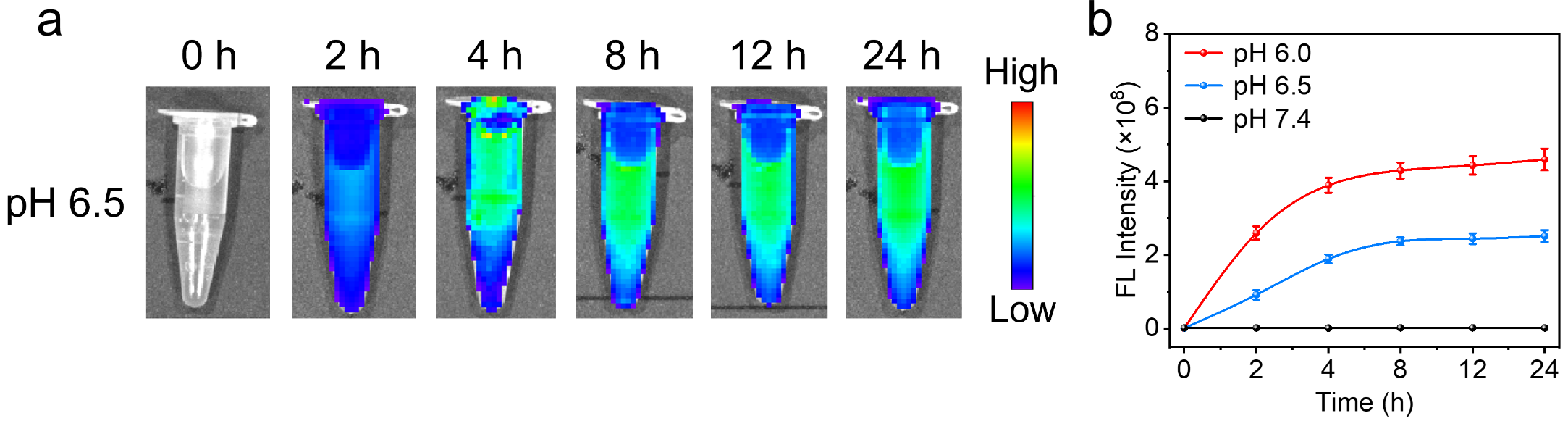


**Figure S7.** (a, b) NIR images and the corresponding quantitative results of Cu-IR783 NPs after incubation of different times at varied pH (6.0, 6.5, and 7.4). Data are presented as the mean ± SD. (n = 3).


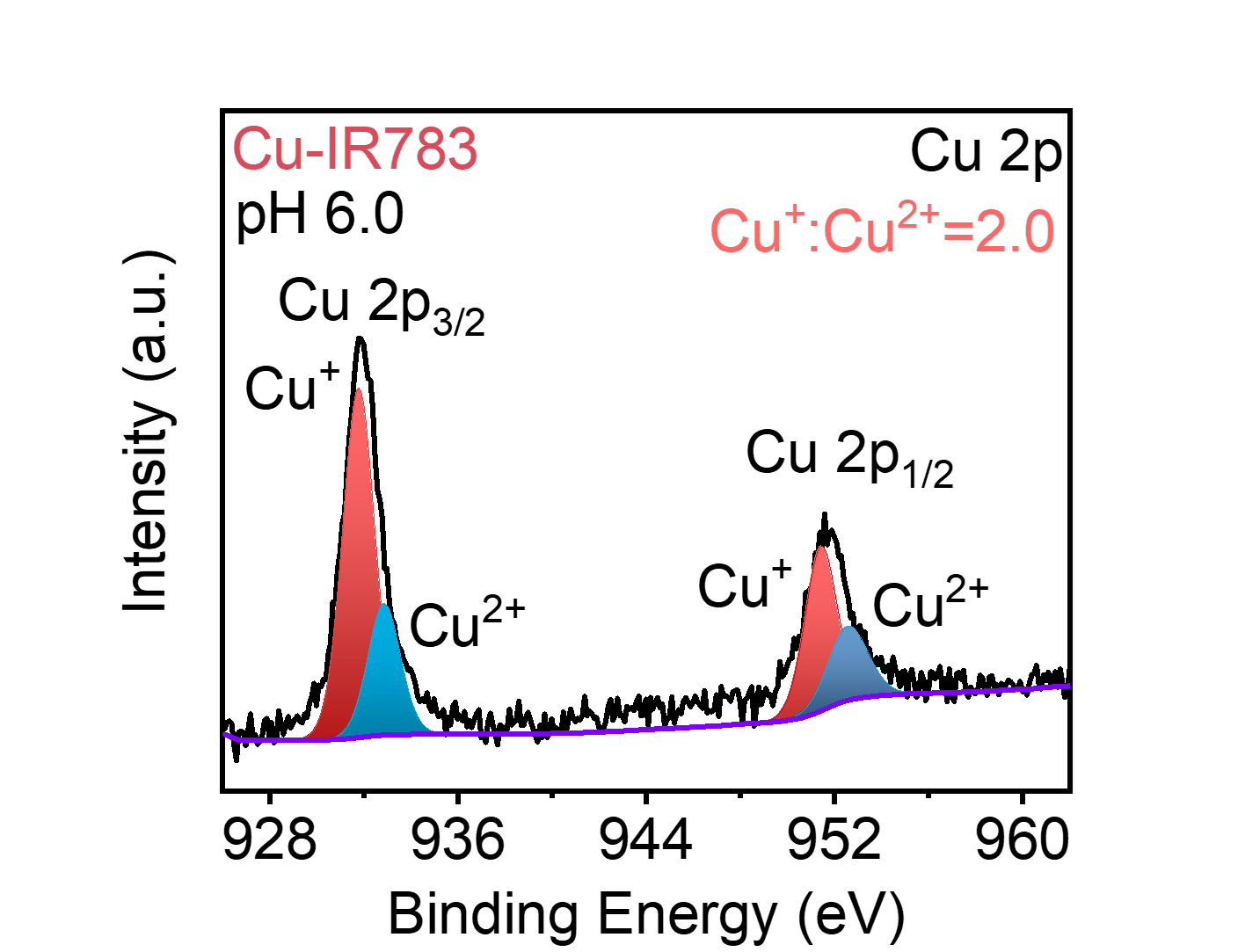


**Figure S8.** High-resolution Cu 2p spectrum of Cu-IR783 after release under acidic conditions.


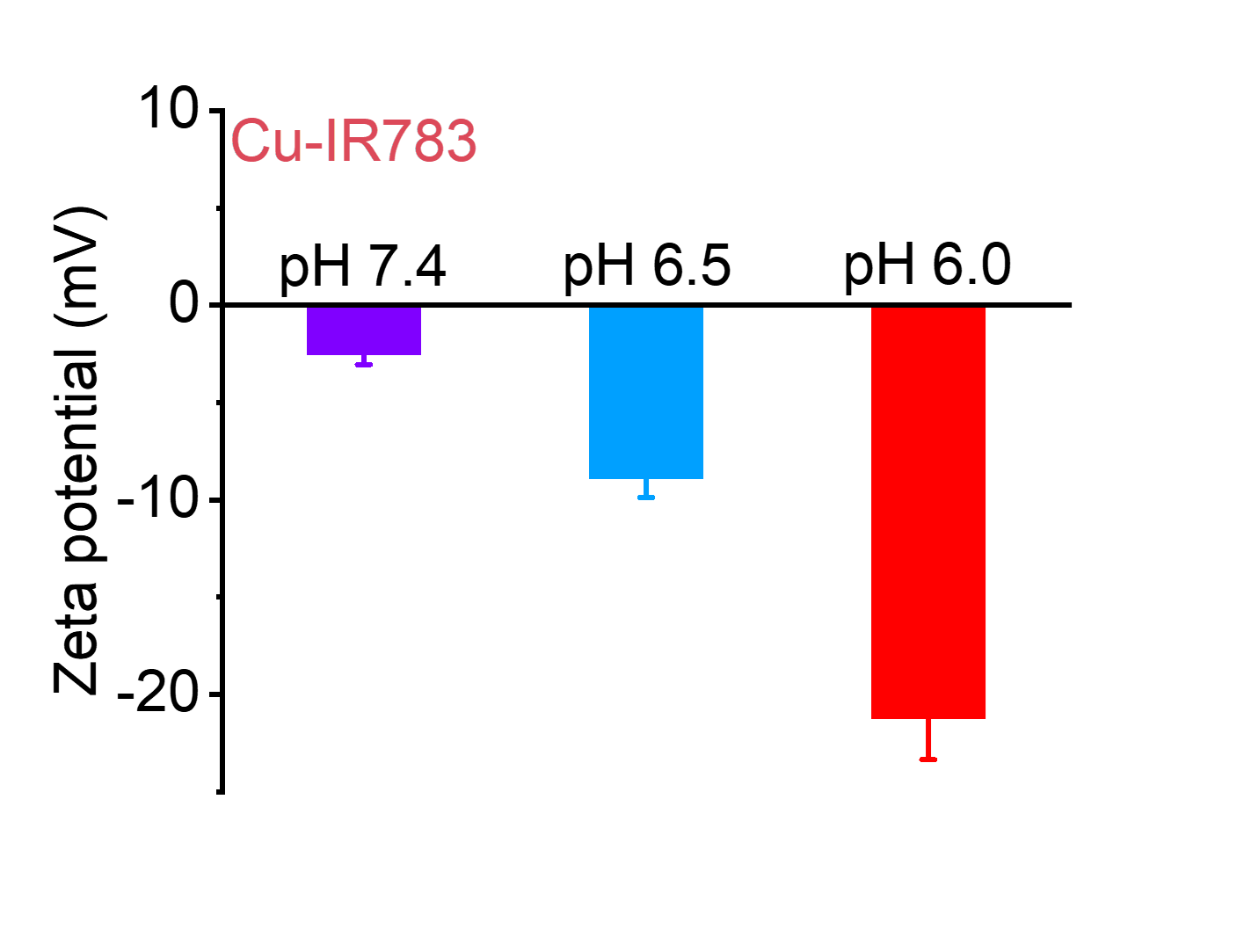


**Figure S9.** (a) Zeta potential of Cu-IR783 NPs after incubation of different times at varied pH (6.0, 6.5, and 7.4). Data are presented as the mean ± SD. (n = 3).


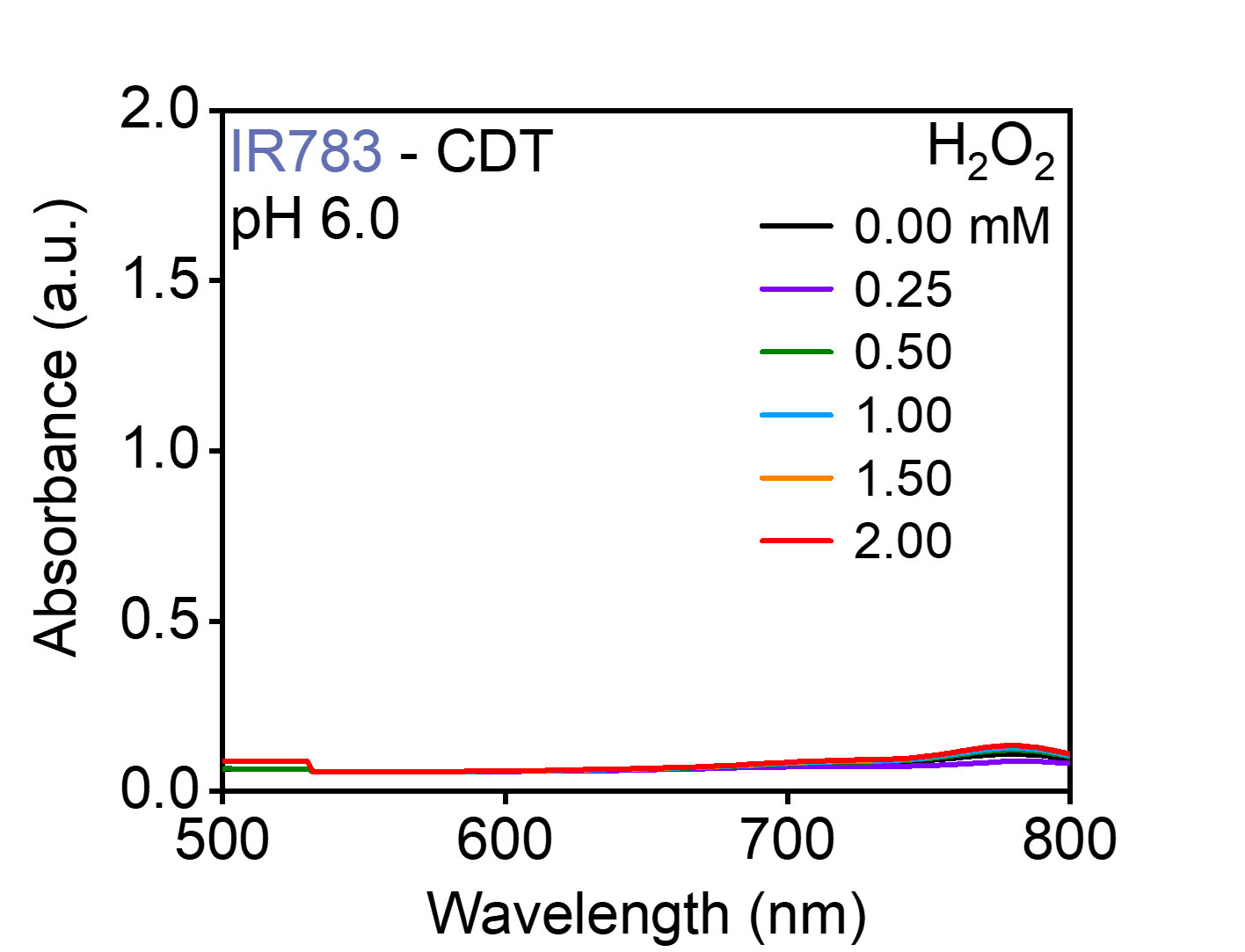


**Figure S10.** Absorption spectra of the free IR783 at pH 6.0 in the presence of H_2_O_2_ with varied concentrations.


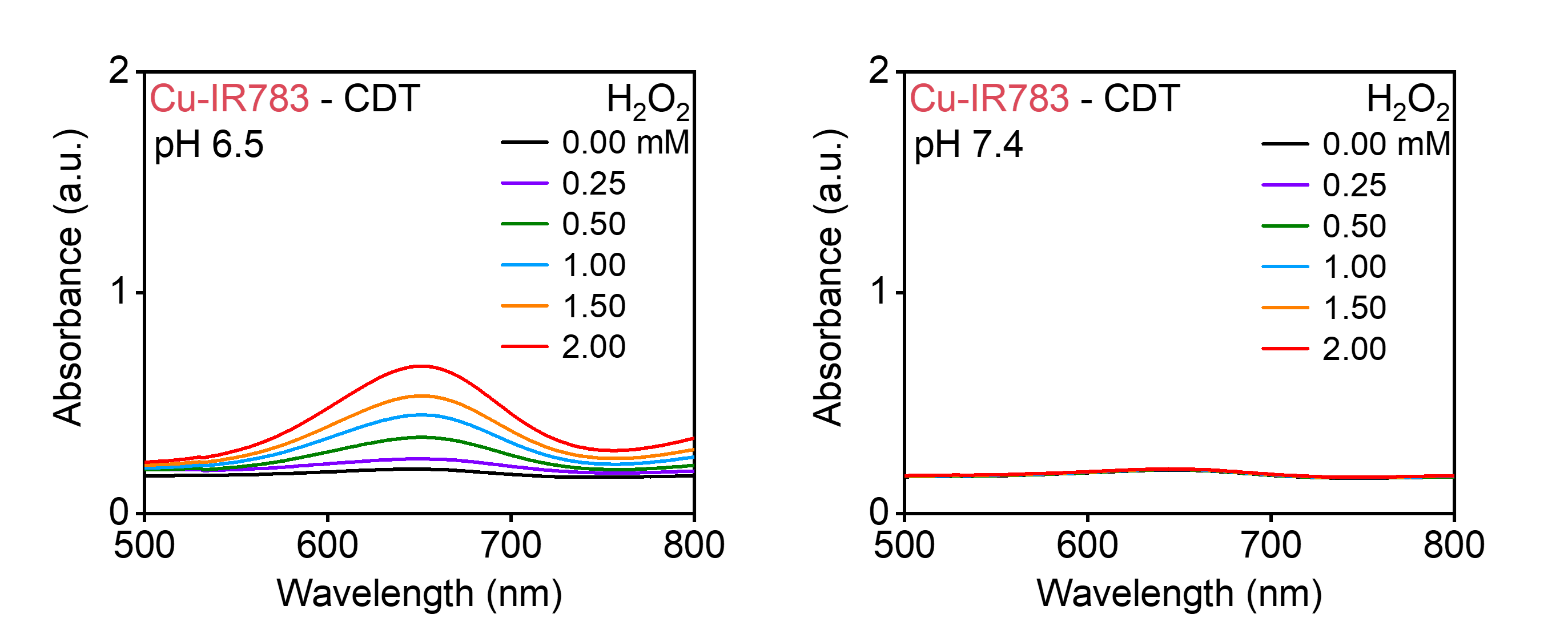


**Figure S11.** Absorption spectra of Cu-IR783 NPs at pH 6.5 and 7.4 in the presence of H_2_O_2_ with varied concentrations.


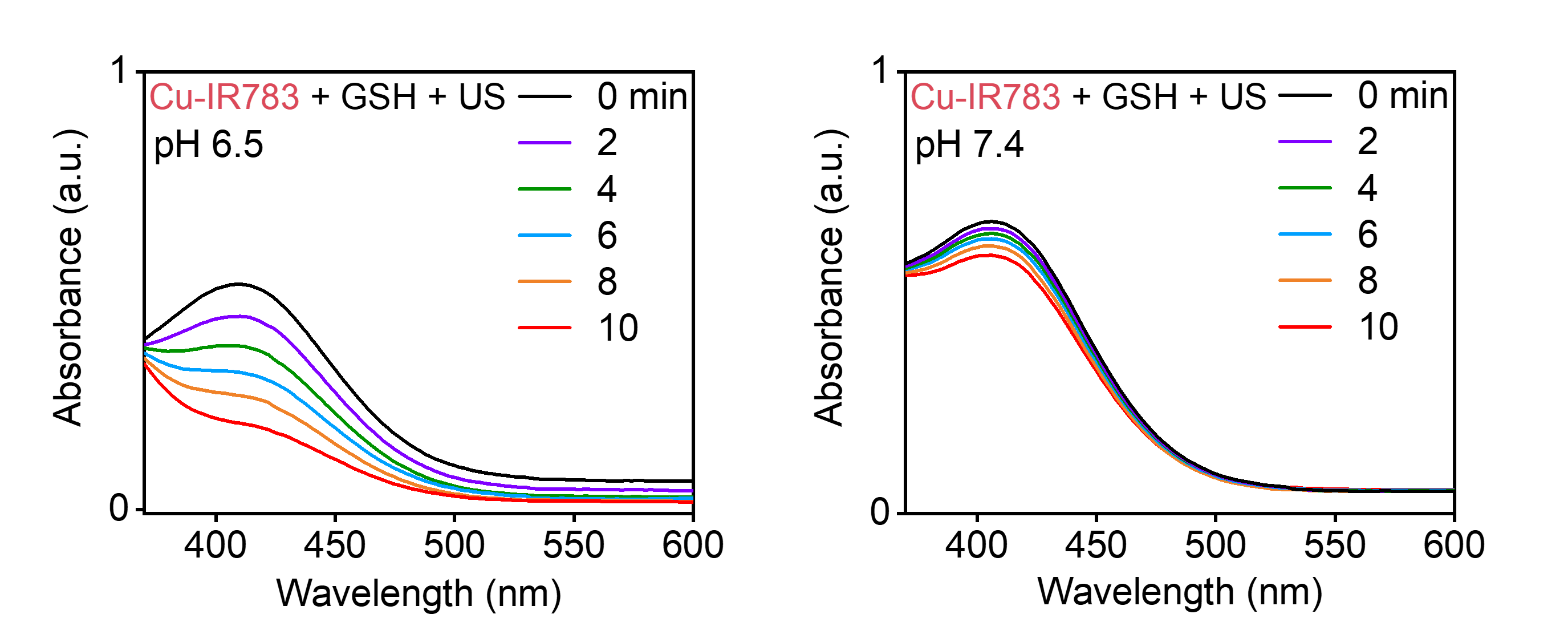


**Figure S12.** Absorption spectra of Cu-IR783 NPs in the presence of GSH and US irradiation at pH 6.5 and 7.4.


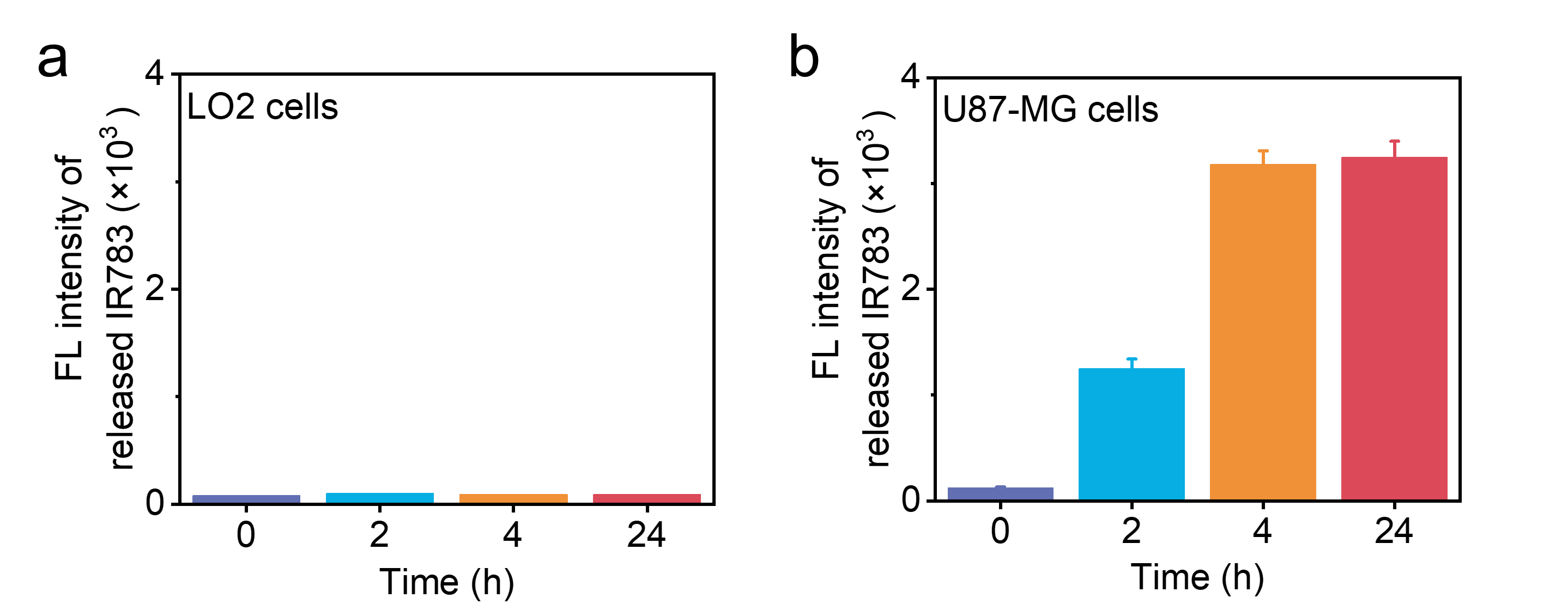


**Figure S13.** The quantitative results of cellular uptake of Cu-IR783 NPs in LO2 (a) and U87-MG (b) cells presented in Figure 4a, b.


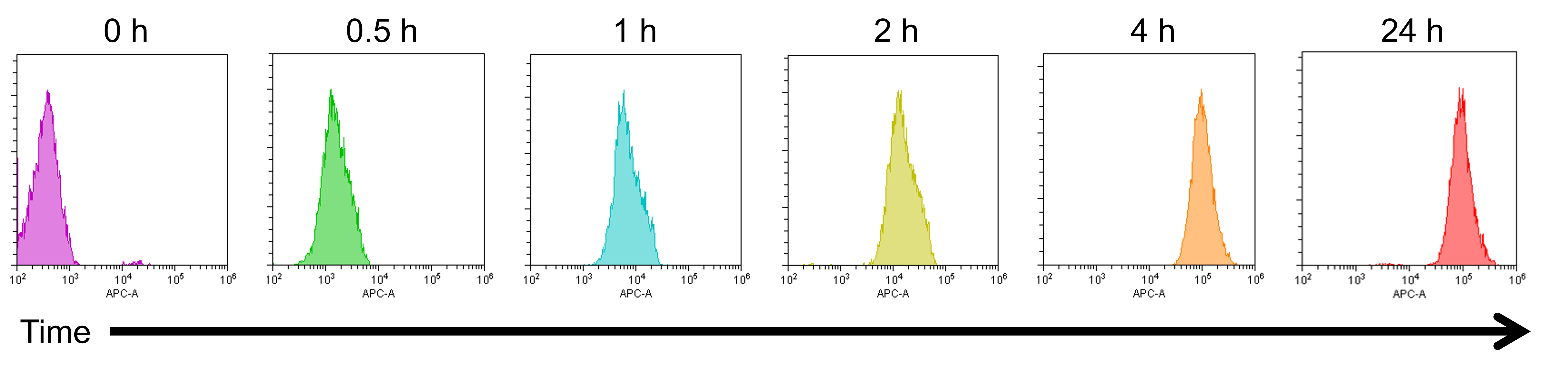


**Figure S14.** Cell uptake of Cu-IR783 NPs after incubation of different times.


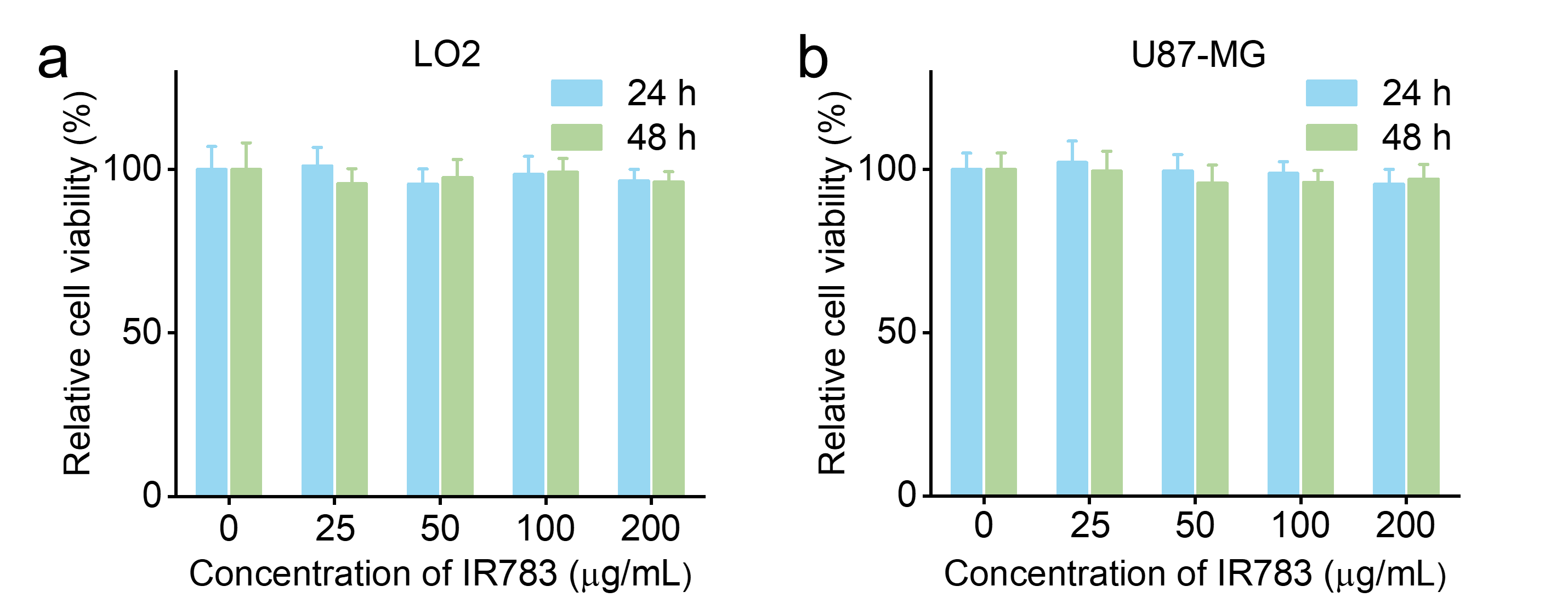


**Figure S15.** (a, b) Cytotoxicity of the free IR783 against LO2 (a) and U87-MG (b). Data are presented as the mean ± SD. (n = 5).


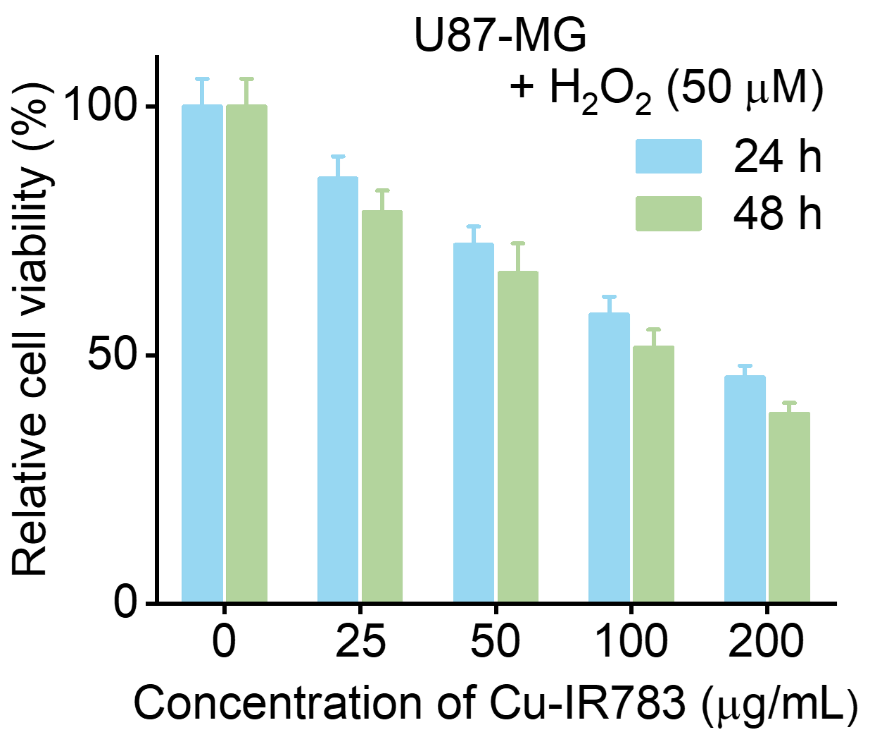


**Figure S16.** Cytotoxicity of the Cu-IR783 against U87-MG in the presence of additional H_2_O_2_ (50 µM). Data are presented as the mean ± SD. (n = 5).


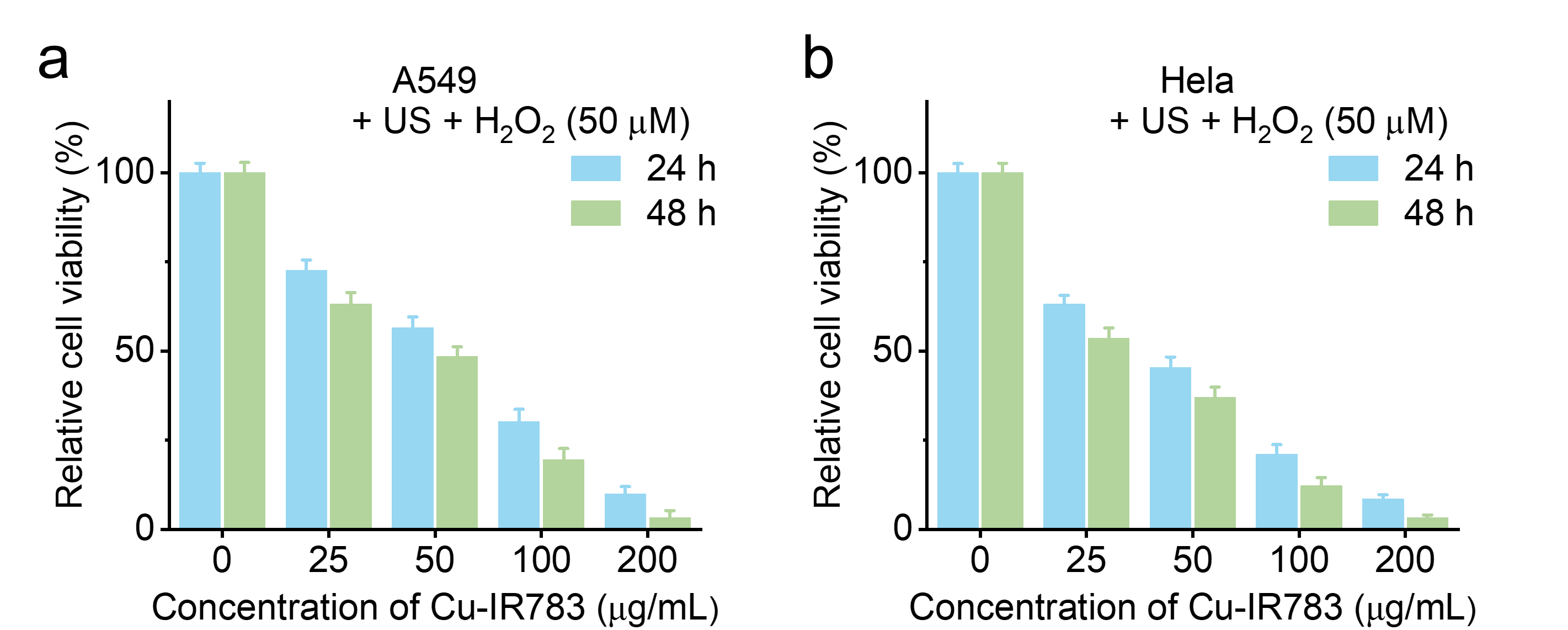


**Figure S17.** Cytotoxicity of the Cu-IR783 against A549 (a) and Hela cells (b) in the presence of additional H_2_O_2_ (50 µM) and US irradiation. Data are presented as the mean ± SD. (n = 5).


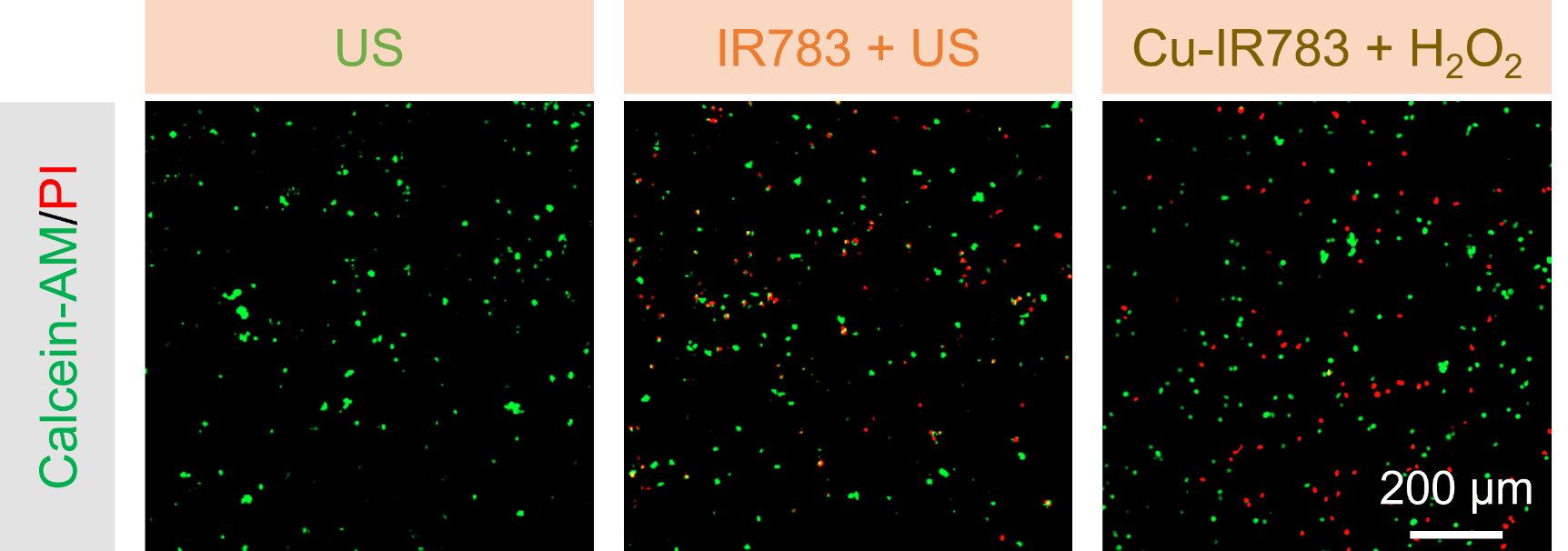


**Figure S18.** Live/dead cell staining of U87-MG cells after different treatments.


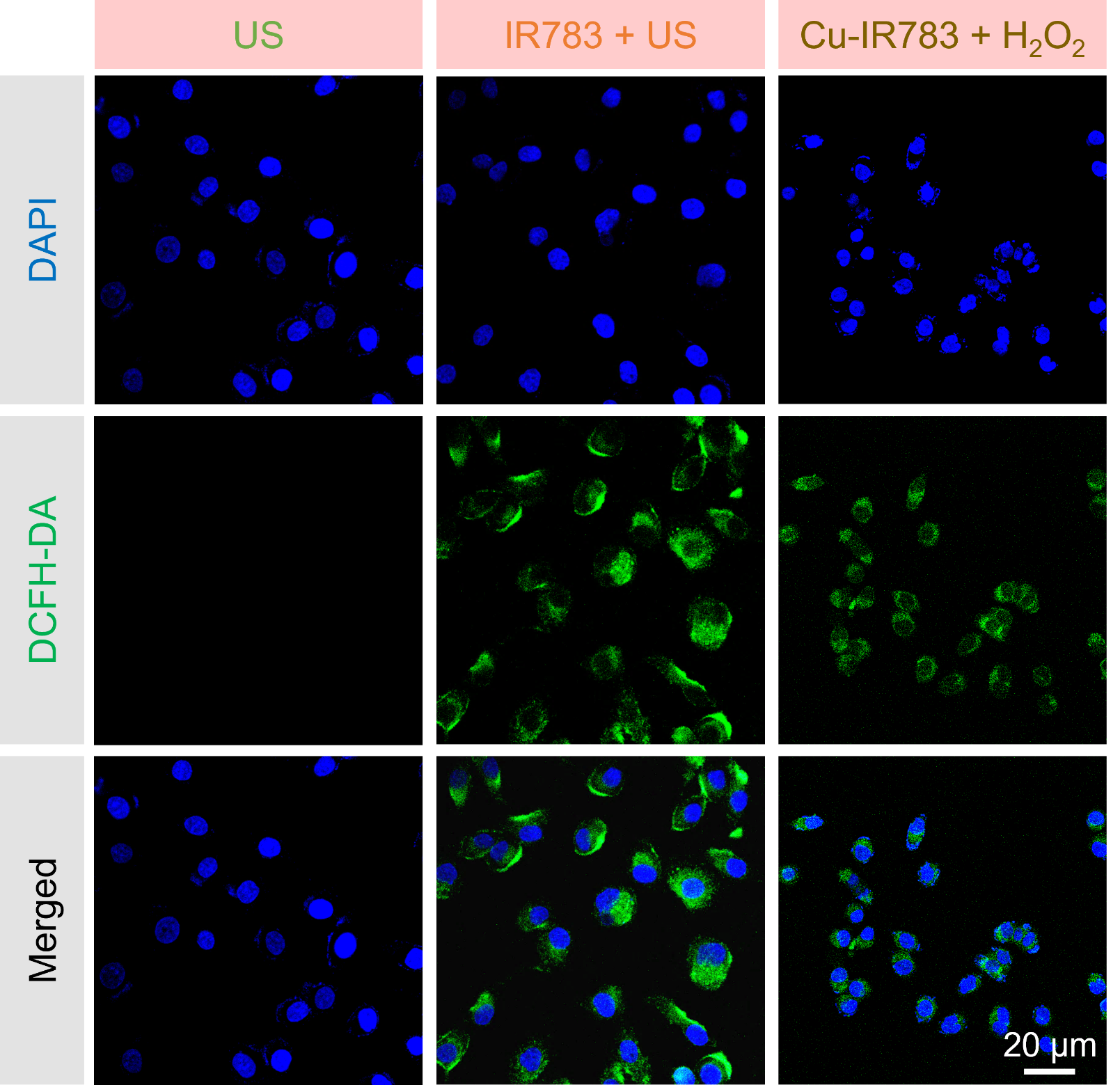


**Figure S19.** ROS staining of U87-MG cells after different treatments.


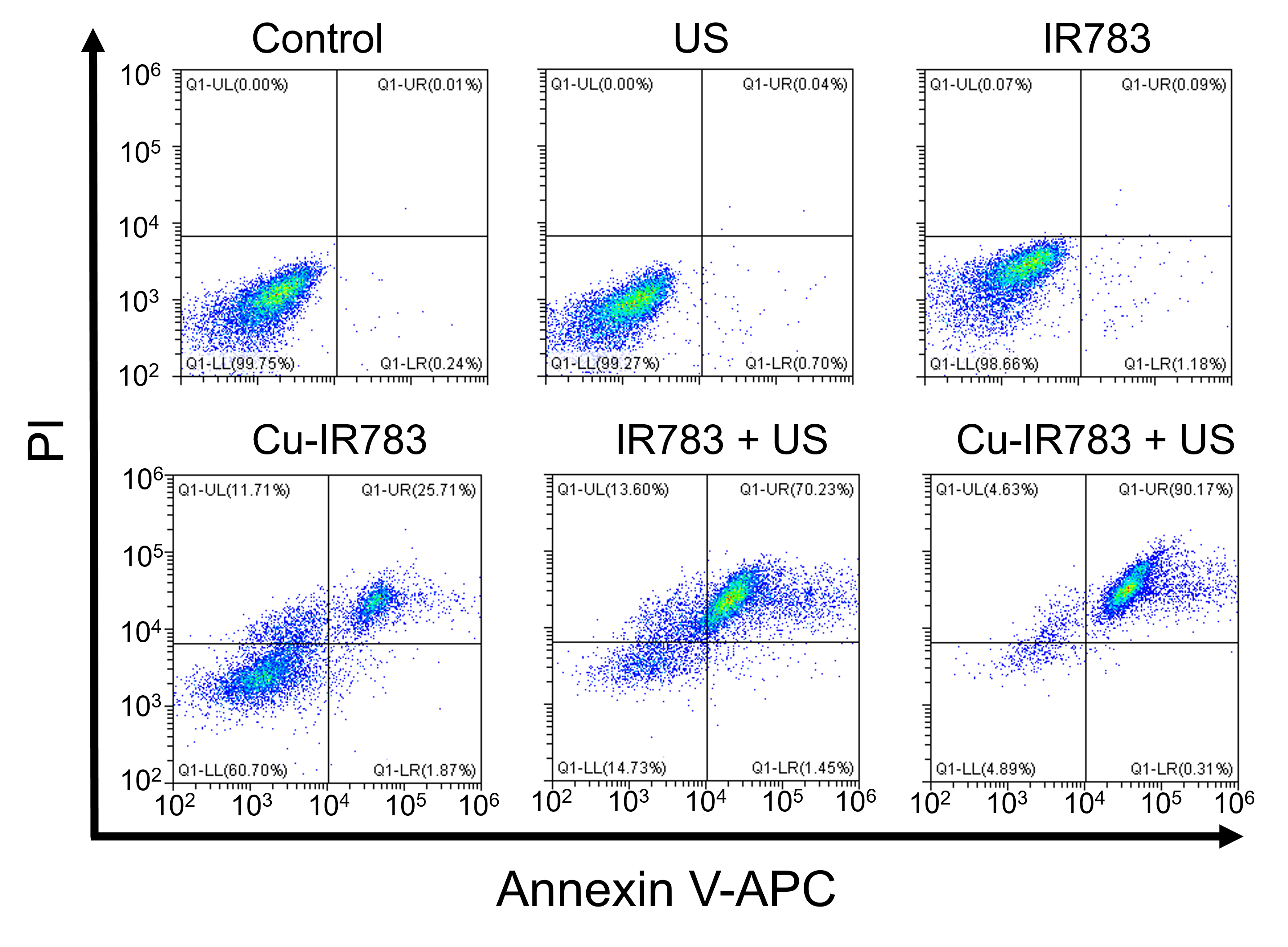


**Figure S20.** Flow cytometry apoptosis assay of U87-MG cells after different treatments.


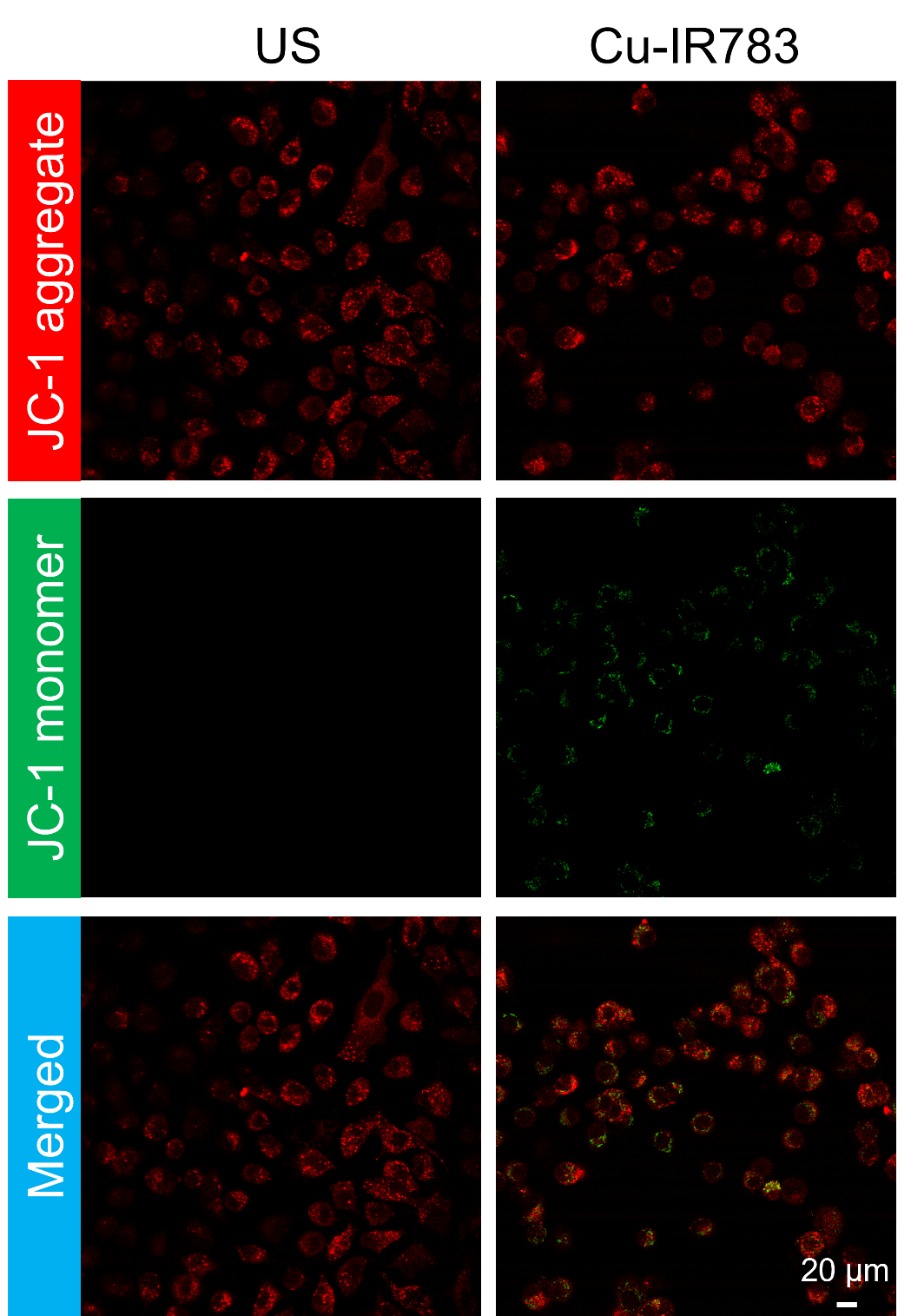


**Figure S21.** Confocal images of 4T1 cells stained with JC-1 after different treatments.


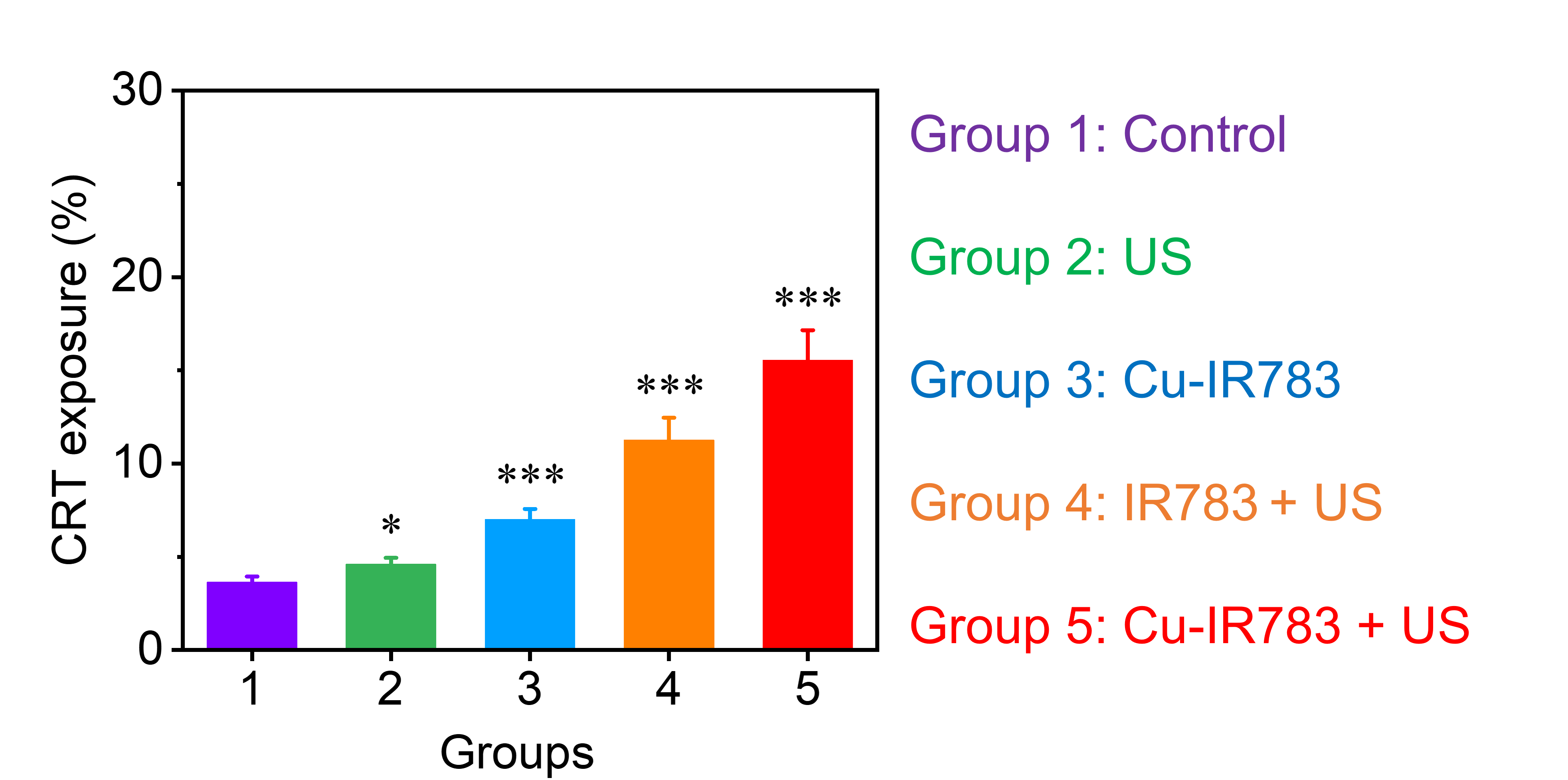


**Figure S22.** The quantitative results of CRT levels in 4T1 cells after different treatments. Statistical significance between the experimental group and the control group is calculated with a two-tailed Student’s t-test. Data are presented as the mean ± SD. (n = 3). *p < 0.05 and ***p < 0.001.


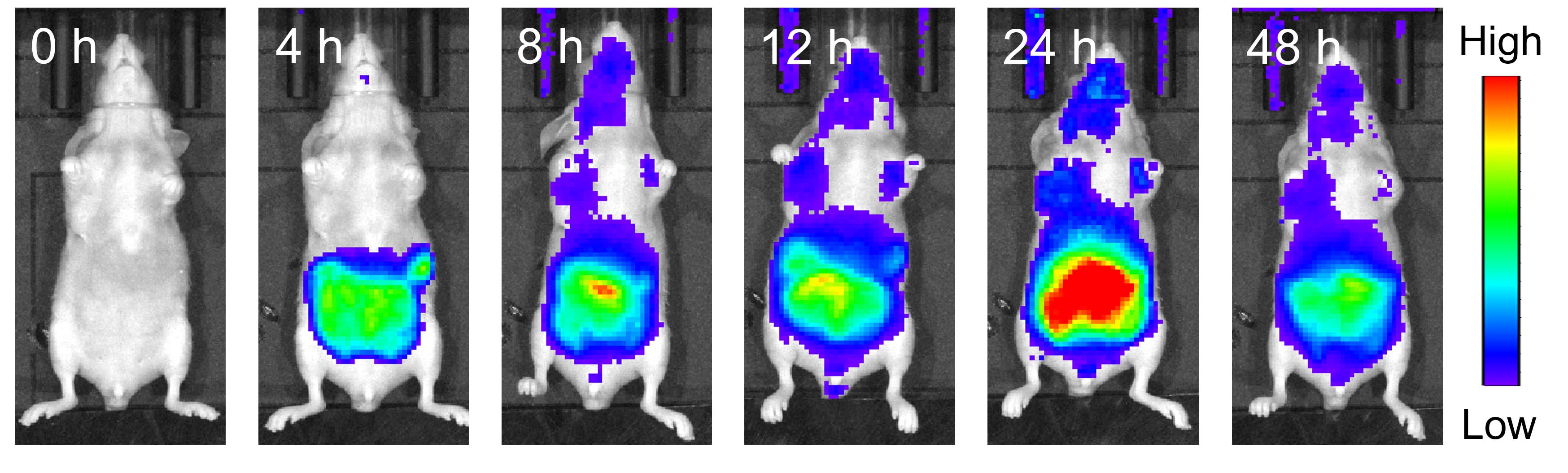


**Figure S23.** NIR imaging of the free IR783 in a subcutaneous tumor model after intravenous injection.


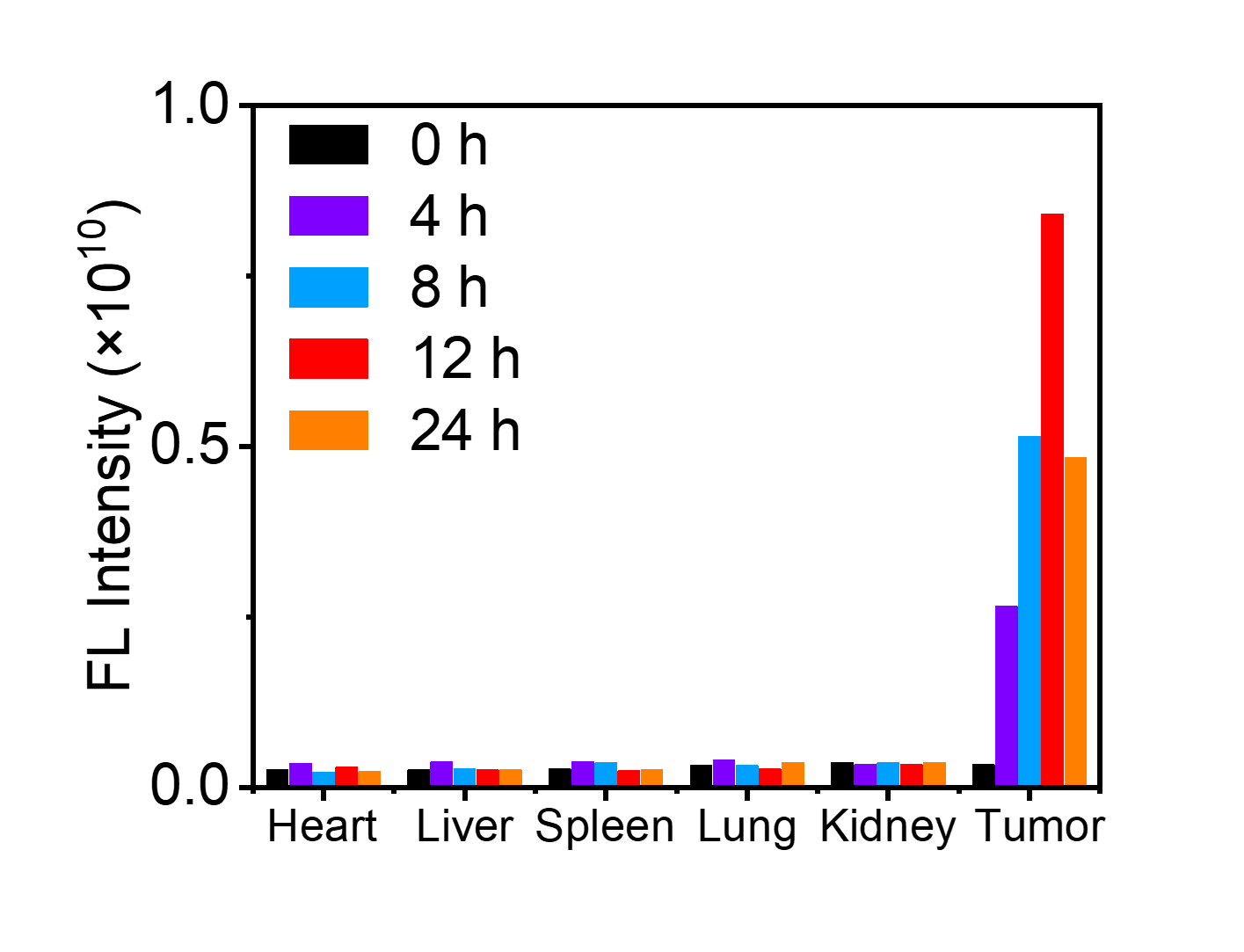


**Figure S24.** The quantitative results of ex vivo NIR imaging presented in Figure 6b.


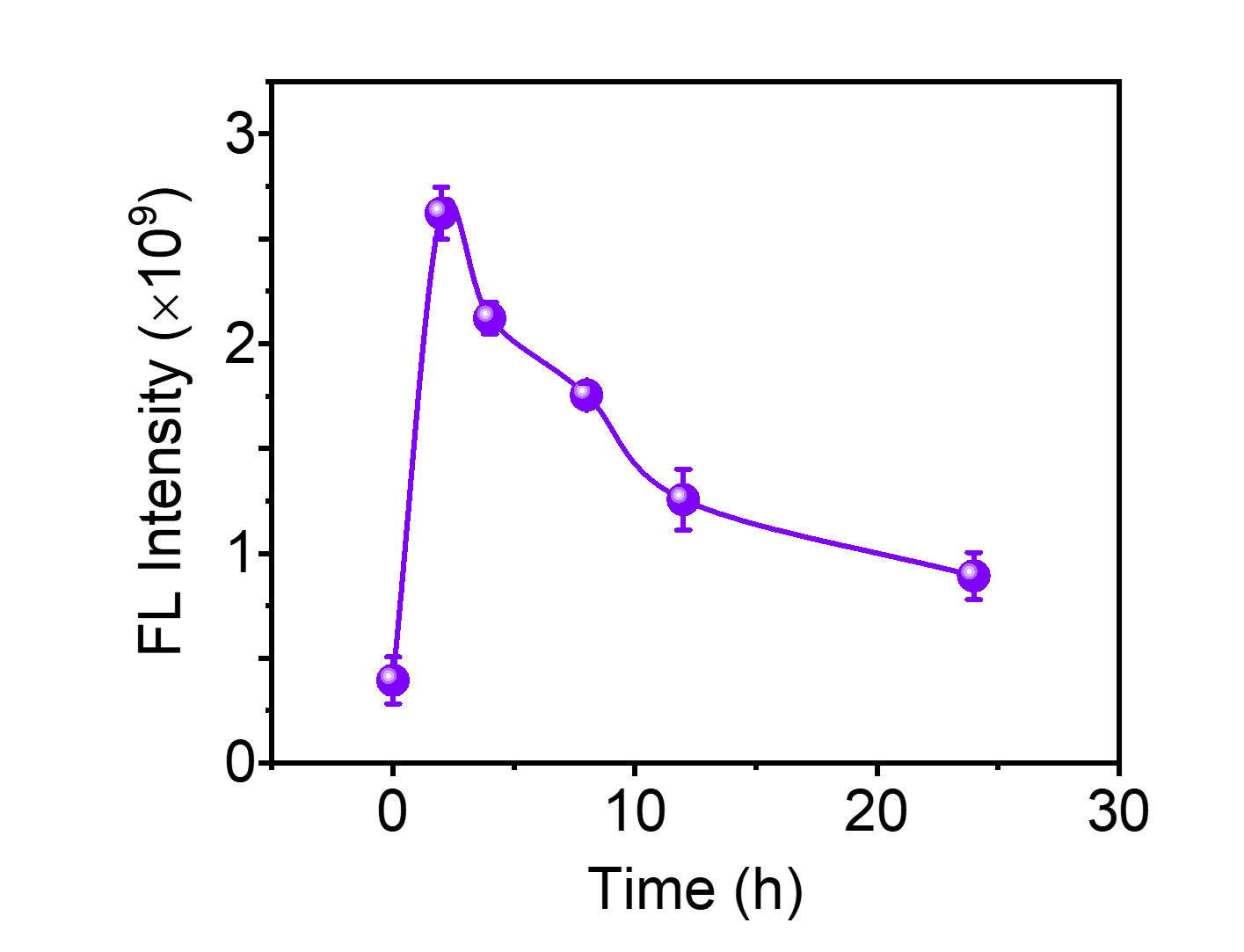


**Figure S25.** The quantitative results of in vivo NIR imaging presented in Figure 6f. Data are presented as the mean ± SD. (n = 3).


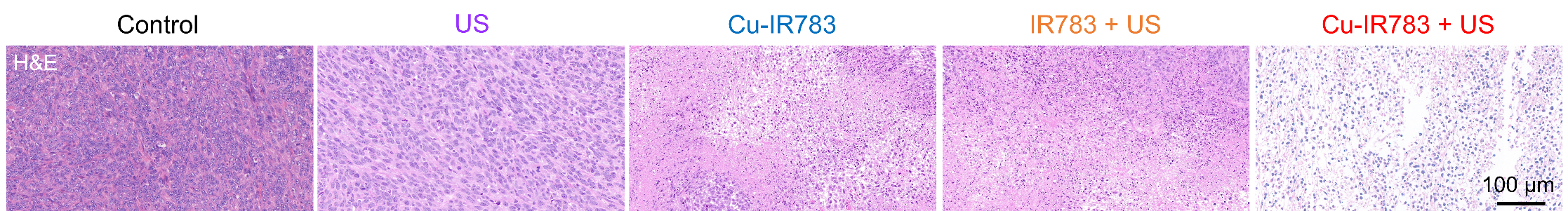


**Figure S26.** H&E staining of tumor tissues in brain of mice after different treatments.


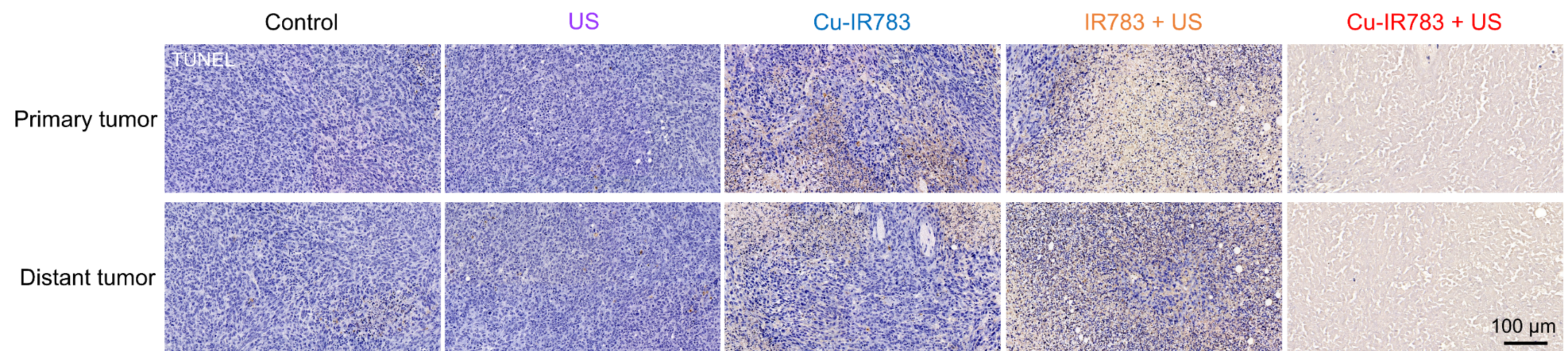


**Figure S27.** TUNEL staining of primary and distant tumor tissues in mice after different treatments.


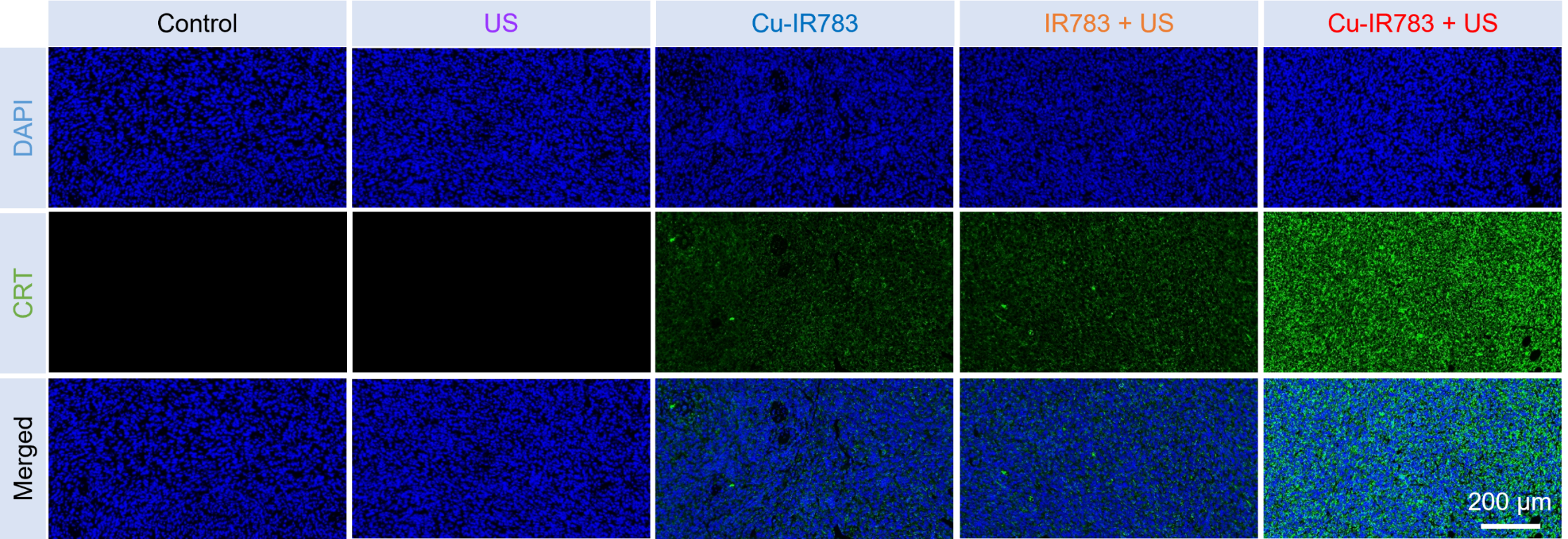


**Figure S28.** CRT levels in tumor tissues of mice after different treatments.


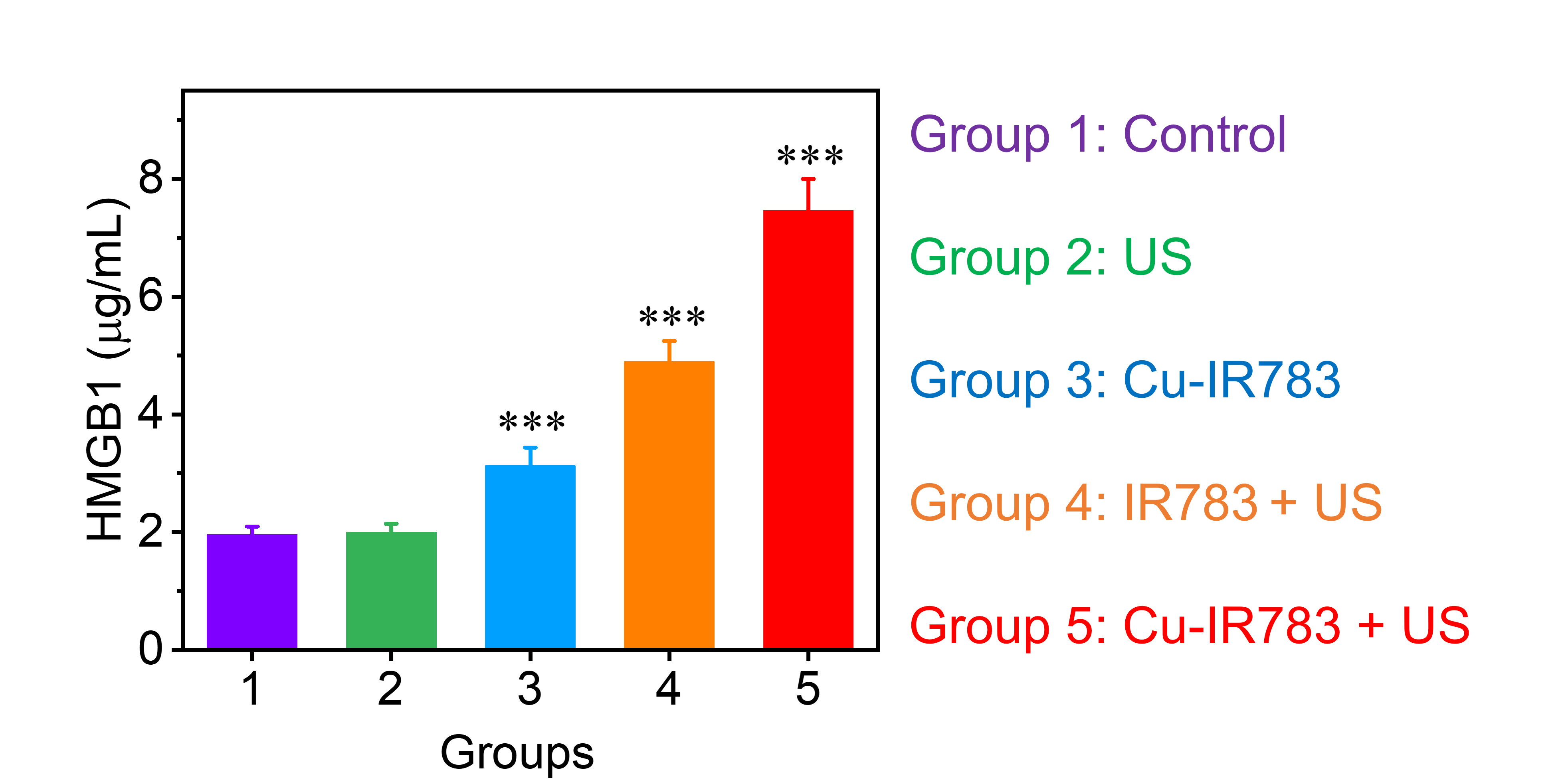


**Figure S29.** HMGB1 levels of tumor tissues of mice after different treatments. Statistical significance between the experimental group and the control group is calculated with a two-tailed Student’s t-test. Data are presented as the mean ± SD. (n = 3). ***p < 0.001.


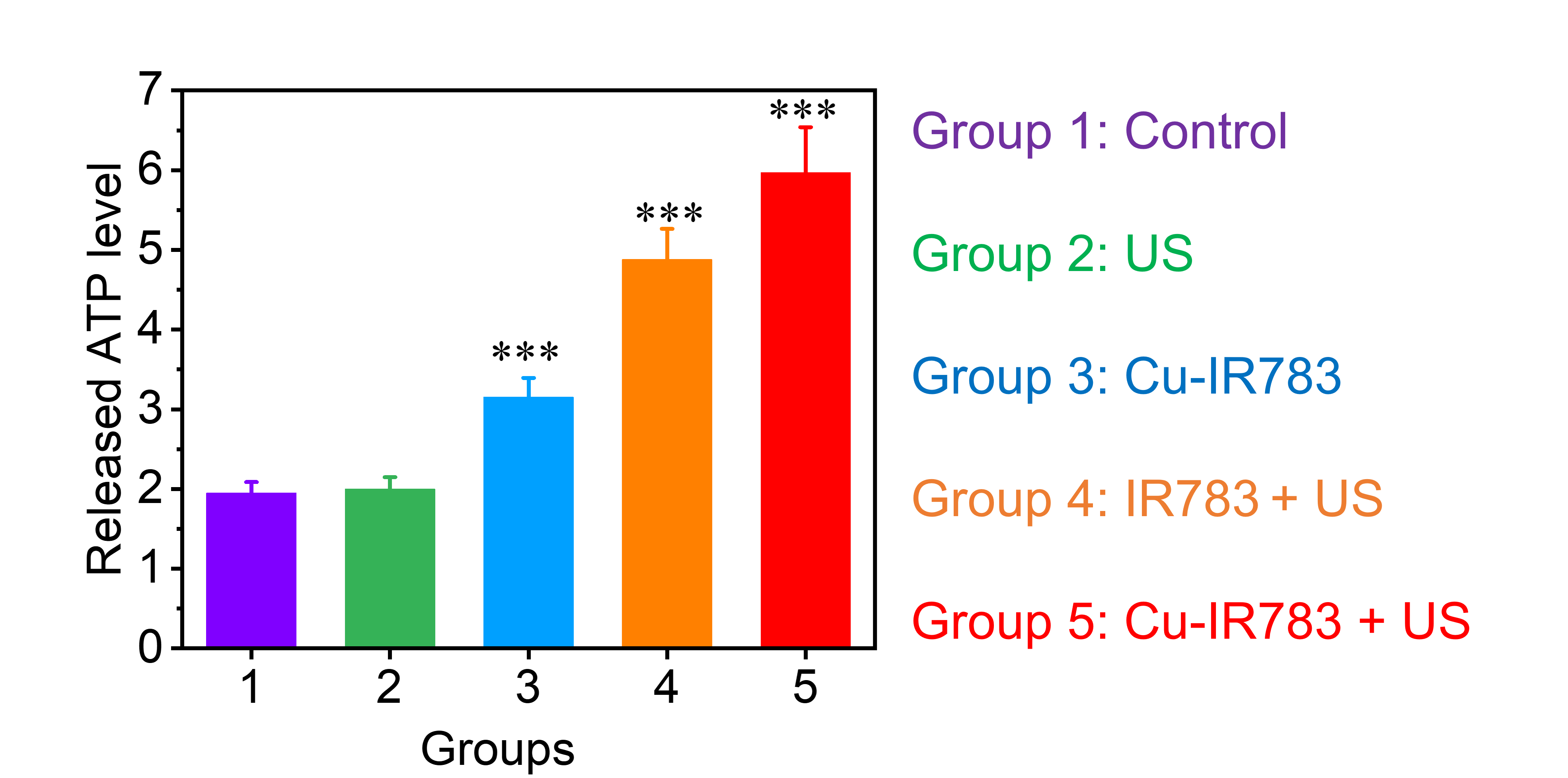


**Figure S30.** ATP levels of tumor tissues of mice after different treatments. Statistical significance between the experimental group and the control group is calculated with a two-tailed Student’s t-test. Data are presented as the mean ± SD. (n = 3). ***p < 0.001.


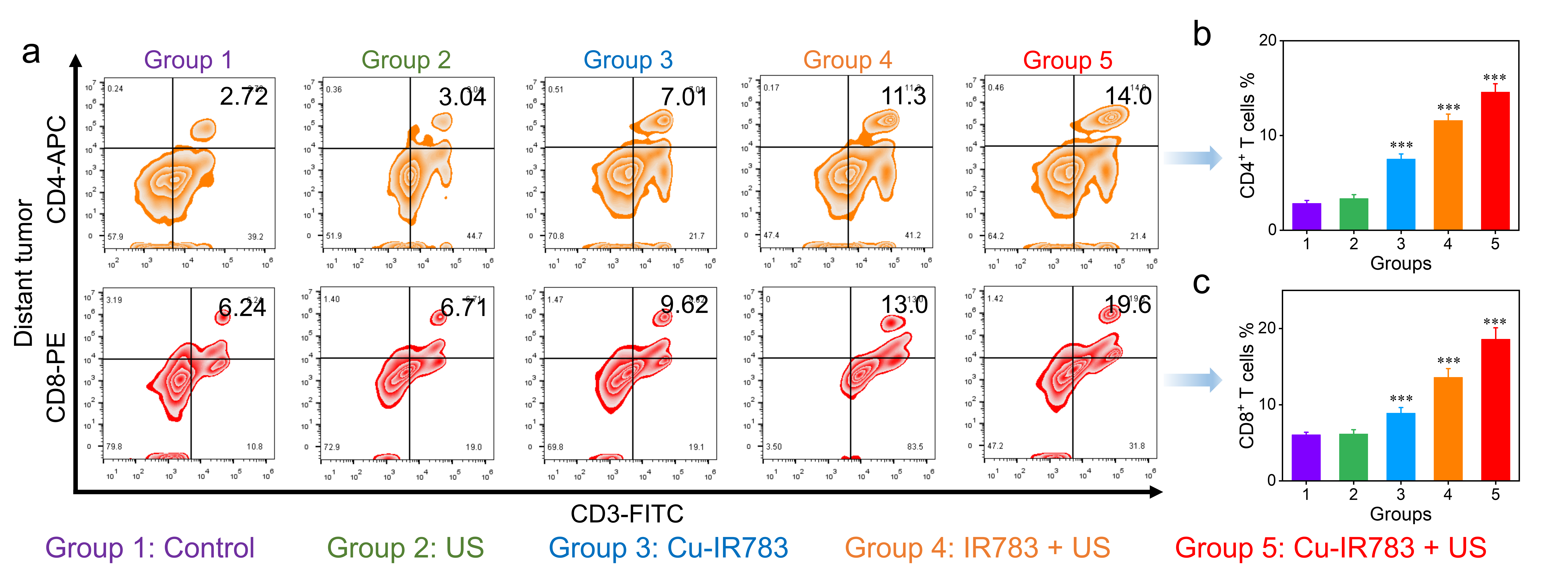


**Figure S31.** Flow cytometry analysis and the corresponding quantification results of the expression of CD4^+^ T cells and CD8^+^ T cells in distant tumors of mice after different treatments (gated on CD3^+^ cells). Statistical significance between the experimental group and the control group is calculated with a two-tailed Student’s t-test. Data are presented as the mean ± SD. (n = 5). ***p < 0.001.


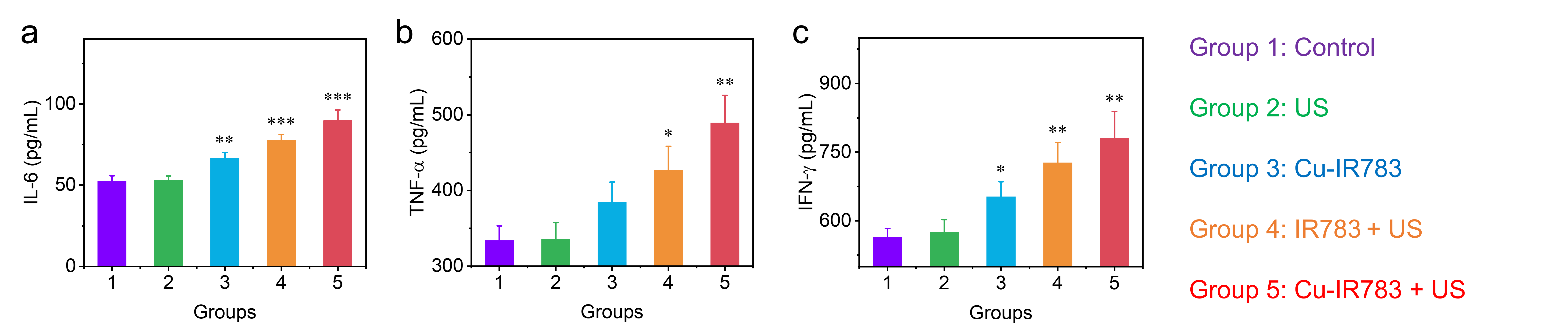


**Figure S32.** Cytokine level of IL-6 (a), TNF-α (b), and INF-γ (c) in blood serum of mice after various treatments. Statistical significance between the experimental group and the control group is calculated with a two-tailed Student’s t-test. Data are presented as the mean ± SD. (n = 5). *p < 0.05, **p < 0.01, and ***p < 0.001.


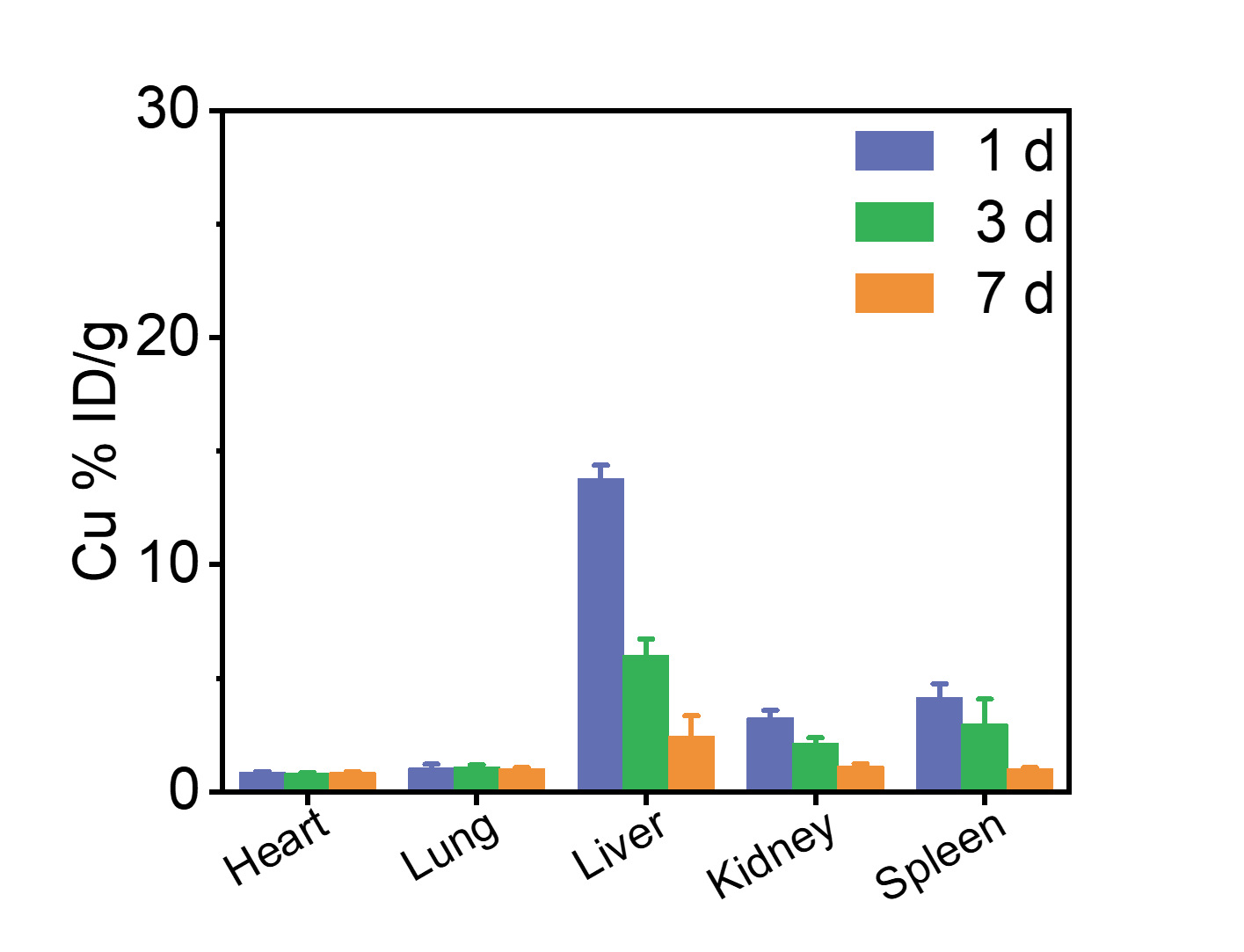


**Figure S33.** Biodistribution of Cu-IR783 post i.v. injection in mice on different days (1, 3, and 7 days).


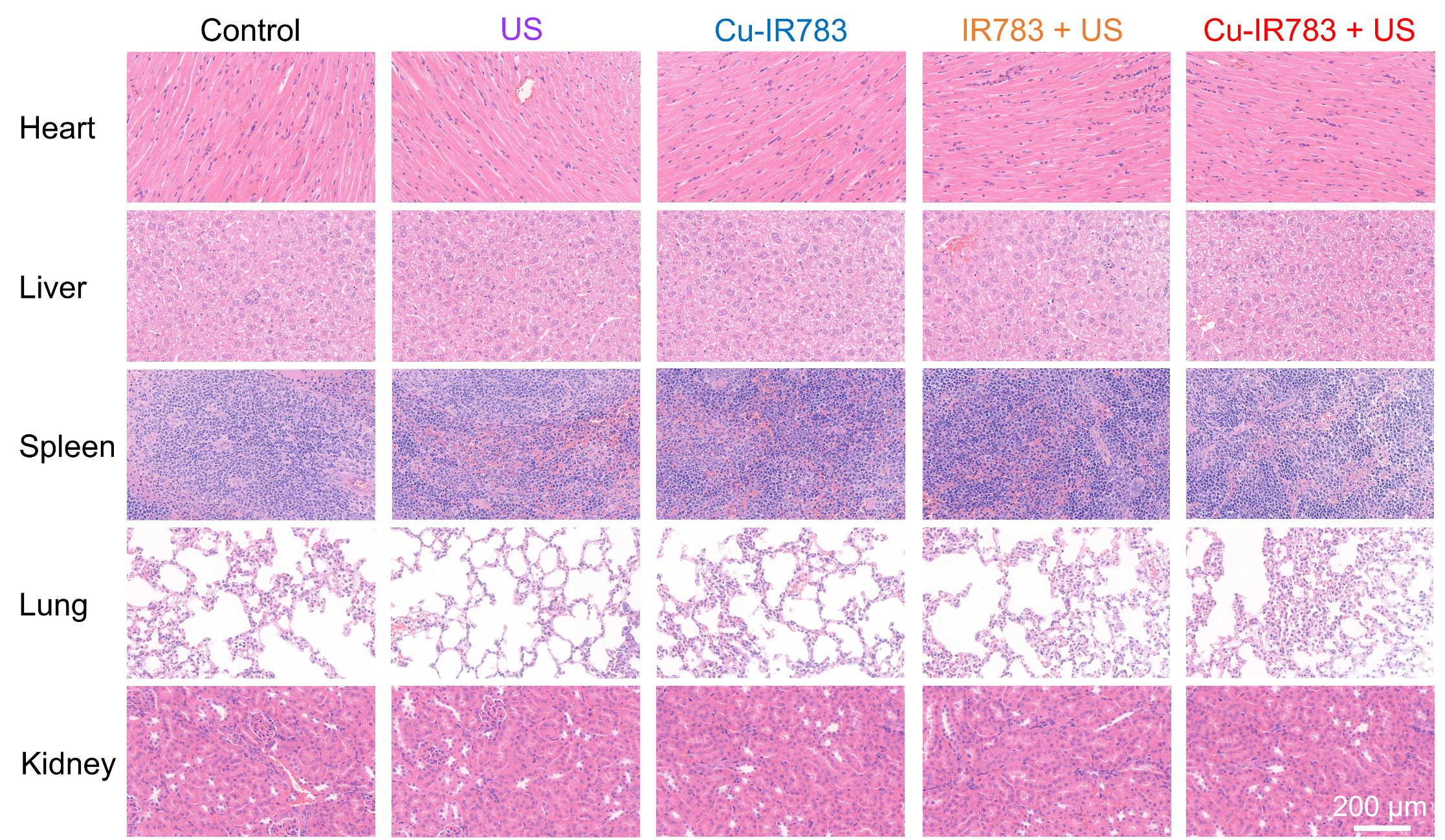


**Figure S34.** H&E-stained images obtained from the major organs (heart, liver, spleen, lung, and kidney) of mice in different treatment groups.


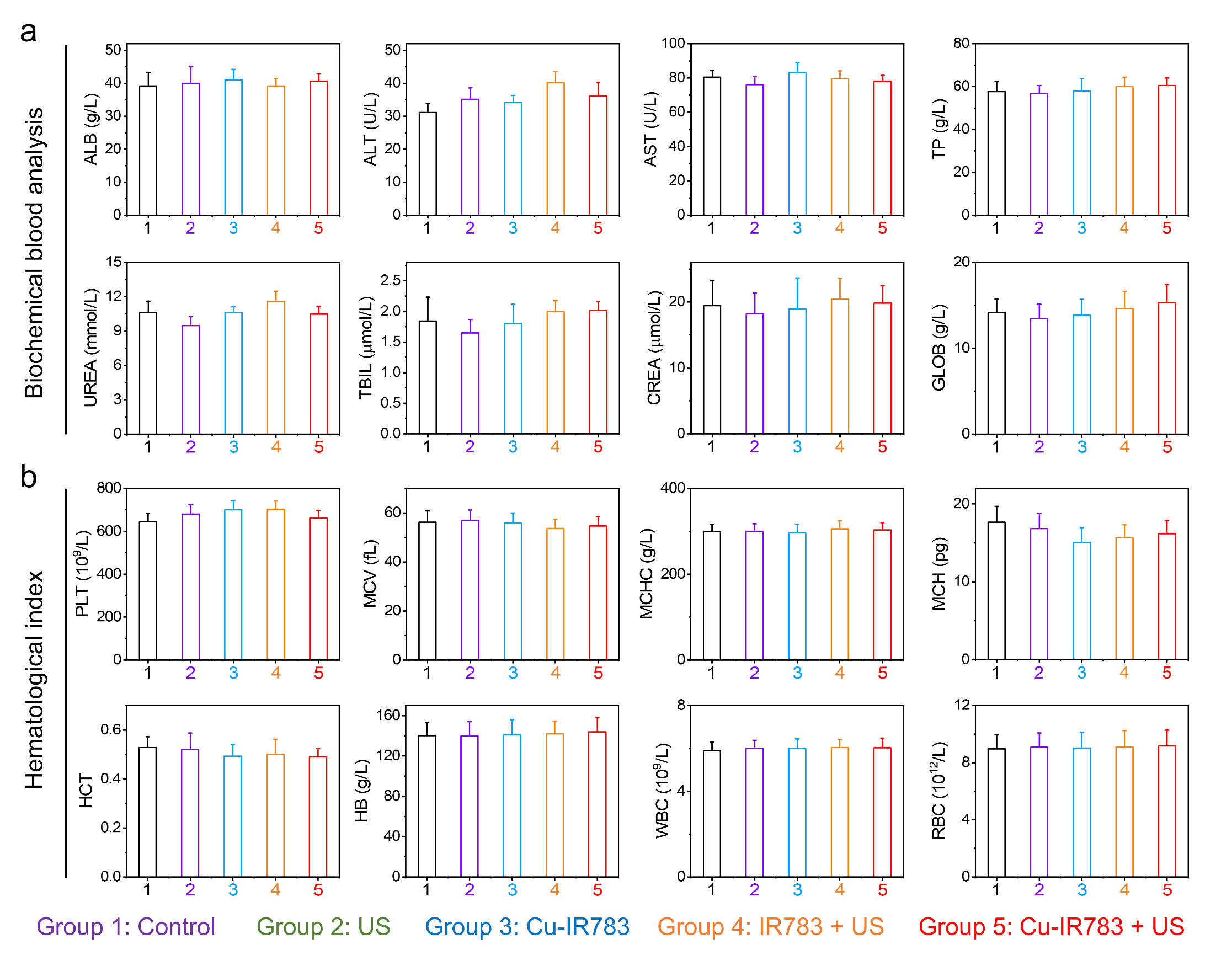


**Figure S35.** (a-b) Biochemical blood analysis (a) and hematological index (b) of the mice that were sacrificed at 18 days after different treatments (n=5 biologically independent samples). The terms of biochemical blood analysis include ALB, ALT, AST, TP, UREA, TBIL, CREA, and GLOB. The terms of hematological index include PLT, MCV, MCHC, MCH, HCT, Hb, WBC, and RBC. Data are presented as the mean ± SD. (n = 5).


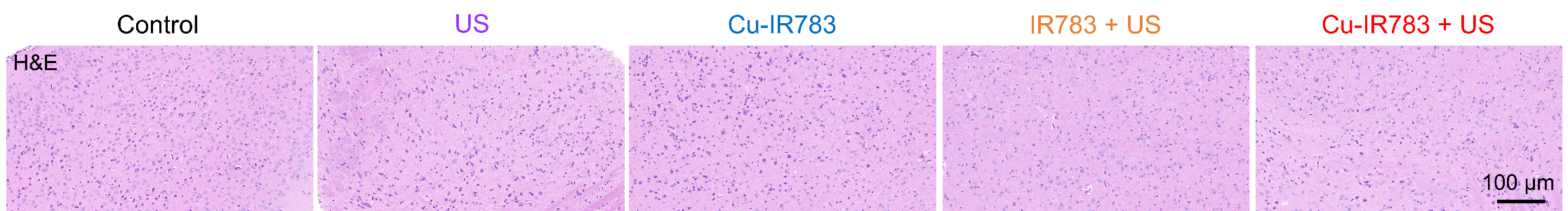


**Figure S36.** H&E staining of brain of mice after different treatments.
